# Spermidine‐Functionalized Injectable Hydrogel Reduces Inflammation and Enhances Healing of Acute and Diabetic Wounds In Situ

**DOI:** 10.1002/advs.202310162

**Published:** 2024-04-11

**Authors:** Qianqian Wu, Runjiao Yang, Wenxuan Fan, Li Wang, Jing Zhan, Tingting Cao, Qiming Liu, Xianshu Piao, Yinghui Zhong, Wenxian Zhao, Shuhan Zhang, Jiaao Yu, Song Liang, Thomas M. Roberts, Bingdi Wang, Zhenning Liu

**Affiliations:** ^1^ Key Laboratory of Bionic Engineering (Ministry of Education) Jilin University Changchun 130022 China; ^2^ Department of Gastroenterology The First Hospital of Jilin University Jilin University Changchun 130021 China; ^3^ Department of Burn Surgery The First Hospital of Jilin University Jilin University Changchun 130061 China; ^4^ Department of Cancer Biology Dana‐Farber Cancer Institute Boston MA 02215 USA; ^5^ Department of Biological Chemistry and Molecular Pharmacology Harvard Medical School Boston MA 02215 USA

**Keywords:** biomaterials, hydrogel, spermidine, inflammation, wound healing

## Abstract

The inflammatory response is a key factor affecting tissue regeneration. Inspired by the immunomodulatory role of spermidine, an injectable double network hydrogel functionalized with spermidine (DN‐SPD) is developed, where the first and second networks are formed by dynamic imine bonds and non‐dynamic photo‐crosslinked bonds respectively. The single network hydrogel before photo‐crosslinking exhibits excellent injectability and thus can be printed and photo‐crosslinked in situ to form double network hydrogels. DN‐SPD hydrogel has demonstrated desirable mechanical properties and tissue adhesion. More importantly, an “operando” comparison of hydrogels loaded with spermidine or diethylenetriamine (DETA), a sham molecule resembling spermidine, has shown similar physical properties, but quite different biological functions. Specifically, the outcomes of 3 sets of in vivo animal experiments demonstrate that DN‐SPD hydrogel can not only reduce inflammation caused by implanted exogenous biomaterials and reactive oxygen species but also promote the polarization of macrophages toward regenerative M2 phenotype, in comparison with DN‐DETA hydrogel. Moreover, the immunoregulation by spermidine can also translate into faster and more natural healing of both acute wounds and diabetic wounds. Hence, the local administration of spermidine affords a simple but elegant approach to attenuate foreign body reactions induced by exogenous biomaterials to treat chronic refractory wounds.

## Graphical abstract

Spermidine‐functionalized hydrogel can mitigate aseptic inflammation induced by exogenous biomaterials and direct more natural healings of acute and diabetic wounds. The aspiration is to mitigate foreign body response by the local administration of spermidine in biomaterials, which may be a game changer for medical implants and even for organ transplantation.

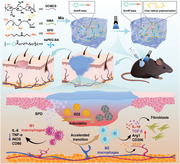

## 1 Introduction

Biomaterials are playing an increasingly important role in tissue engineering and implantable medical devices.^[^ ^1^ ^]^ However, exogenous biomaterials may trigger a series of cellular and molecular events in the host immune system, leading to inflammatory responses and even rejection of medical devices.^[^ ^2^ ^]^ Indeed, inflammation has been widely recognized as a key factor affecting regeneration.^[^ ^3^ ^]^ Hence, anti‐inflammatory agents, such as dexamethasone and heparin, have been used to endow immunomodulatory functions to implanted biomaterials.^[^ ^4^ ^]^ Yet, the application of these drugs often leads to adverse reactions and high costs.^[^ ^5^ ^]^

Spermidine (SPD) is a natural polyamine originally identified in semen. Recently, it has been reported that the level of SPD is significantly lower in the semen of infertile men; thus, it has been speculated that SPD can regulate the female immune system to protect sperms, as “foreigners” in the woman's body, to facilitate fertilization.^[^ ^6^ ^]^ Consistent with this hypothesis, animal experiments have shown that SPD can improve the success rate of embryo implantation and development.^[^ ^7^ ^]^ More intriguingly, several recent works have demonstrated a broader immunomodulatory function for SPD, such as anti‐inflammatory effects^[^ ^8^ ^]^ and improvement in anti‐tumor immunity.^[^ ^9^ ^]^ Hence, we have envisioned that the local administration of SPD in biomaterials can reduce the inflammation induced by exogenous biomaterials and promote wound healing, thus avoiding the undesirable adverse reactions caused by immunosuppressive drugs.

Wound healing is a highly coordinated process that can be divided into 4 different stages: hemostasis, inflammation, proliferation, and remodeling.^[^ ^10^ ^]^ The inflammatory response plays a critical role in wound healing and unresolved inflammation stalls the progression of healing, resulting in chronic or even non‐healing wounds.^[^ ^11^ ^]^ Indeed, the chronic non‐healing wound is one of the most prevalent complications of diabetes.^[^ ^12^ ^]^ Underlying chronic diabetic wounds is a complex micro‐environment, wherein persistent inflammation, the accumulation of advanced glycation end products, and excessive oxidative stress lead to microvascular complications, neuropathy, and cellular senescence in the local wound area.^[^ ^5^, ^10^, ^12^, ^13^ ^]^

Hydrogels engineered to imitate natural extracellular matrix (ECM) are promising biomaterials to facilitate wound healing and injectable self‐healing hydrogels can be quickly injected into irregular or deep wounds.^[^ ^14^ ^]^ Moreover, injectable hydrogels can not only be used in a minimally invasive manner but also be directly bio‐printed at the target location.^[^ ^15^ ^]^ However, injectable hydrogels usually suffer from poor mechanical properties due to weak noncovalent interactions.^[^ ^14^, ^16^ ^]^ In addition, most injectable hydrogels undergo rapid degradation after application, and, thus, cannot provide long‐term biological function.^[^ ^17^ ^]^ Hence, we have designed a double network hydrogel based on both reversible cross‐linking of dynamic imine bonds and irreversible cross‐linking of nondynamic covalent bonds to achieve both an excellent mechanical strength for bio‐printing and a desirable degradation rate for sustained action.

**Scheme 1:**
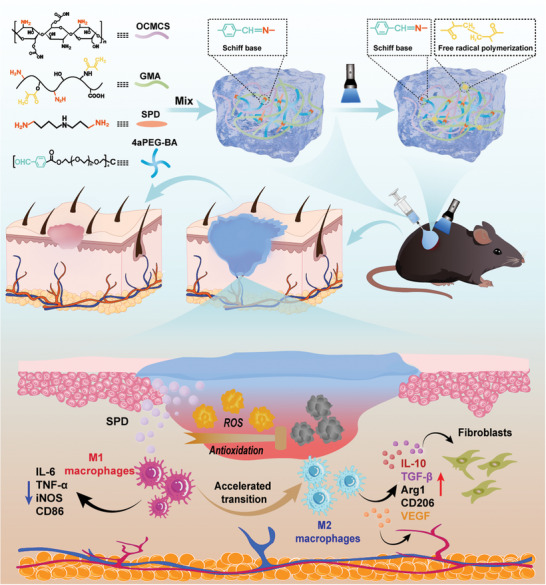
Schematic illustration of the preparation of DN‐SPD hydrogel and its immunomodulatory function in wound healing.

Previously, we have fabricated two types of SPD‐functionalized biomaterials, i.e. SPD‐functionalized hydrogels and composite films, and found both can inhibit the implant‐mediated inflammatory response and enhance the healing of skin wounds.^[^ ^18^ ^]^ Recent work from other groups has also demonstrated that SPD‐containing hydrogels can yield better regeneration of nervous tissue^[^ ^19^ ^]^ and periodontal ligaments.^[^ ^20^ ^]^ In this work, we demonstrate the development of an SPD‐empowered injectable hydrogel for bio‐printing and examine the immunomodulatory effect of SPD on the healing of more complicated skin wounds, i.e. chronic diabetic wounds (**Scheme** **1**).

## 2 Results

### 2.1 Preparation and Characterization of Hydrogels

SPD‐functionalized double network hydrogels (DN‐SPD) were prepared from a mixture of *O*‐carboxymethyl chitosan (OCMCS), gelatin methacryloyl (GMA), and SPD. First, OCMCS/GMA/SPD solutions with different concentrations of SPD were mixed with the crosslinker solution of 4‐arm poly(ethylene glycol) benzaldehyde (4aPEG‐BA) at room temperature to prepare single network hydrogels (SN) via Schiff base reaction (Figure S1a, Supporting Information). The final concentration of 4aPEG‐BA in SN hydrogels is 0.50% (m/v). Then the GMA in SN hydrogels was photo‐crosslinked under blue light (405 nm) to form a second covalent network. It should be noted that the first network formed by imine bonds is dynamic and thus injectable as shown below, whereas the second network based on free radical polymerization is nondynamic. In parallel, double network hydrogels using diethylenetriamine (DETA) in place of SPD were also synthesized and denoted as DN and DN‐DETA hydrogels, respectively. DETA closely resembles SPD in chemical structure (Figure S1b, Supporting Information) and thus enables an “operando” comparison in biological experiments. The numbers at the end of the names of DN‐SPD and DN‐DETA hydrogels indicate the corresponding micromolar (µM) concentrations of SPD and DETA in the hydrogels. The chemical bonds of the hydrogels were verified by Fourier transform infrared spectroscopy (FI‐TR). The bands at 1645 and 1534 cm^−1^ can be assigned to C═N and C‐C stretching vibrations, respectively (Figure S1c, Supporting Information).

**Figure 1:**
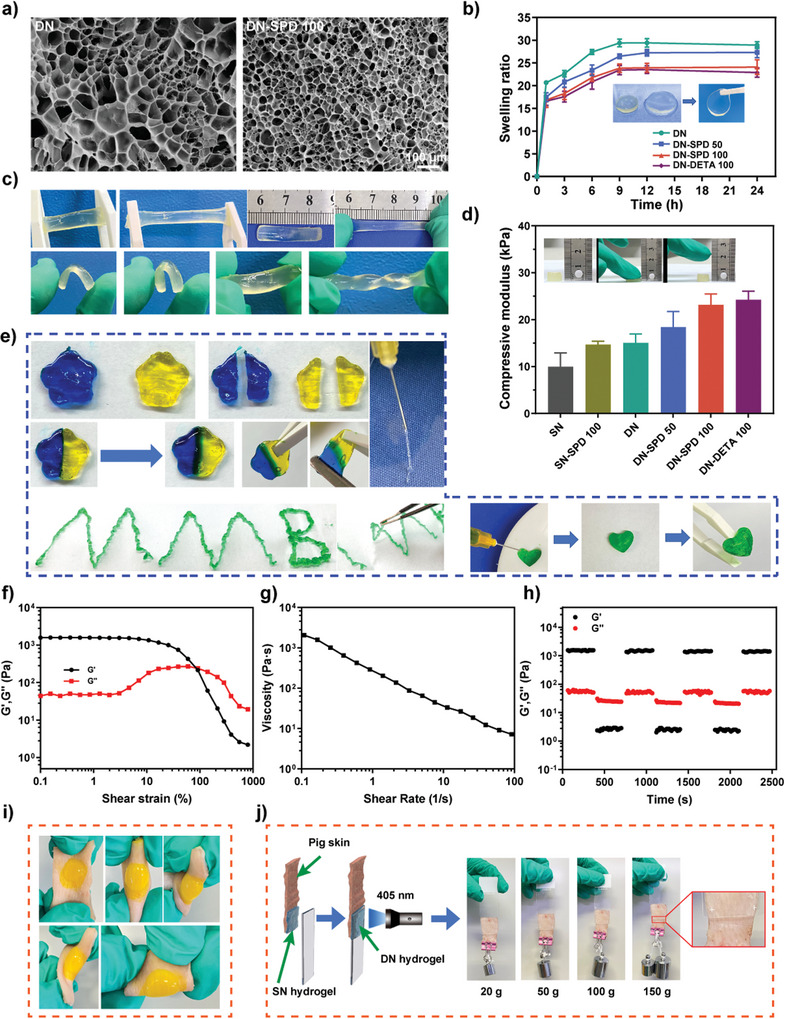
Physical characterizations of hydrogels. a) SEM images of DN and DN‐SPD 100 hydrogels. b) Swelling ratios (*n* = 3). c) Photographs of DN‐SPD 100 hydrogels withstanding stretching, bending, and twisting. d) Compressive moduli of different hydrogels (*n* = 3). e) Photographs showing self‐healing, injectability, and conformity of SN‐SPD 100 hydrogels. f‐h) Rheological analysis of SN‐SPD 100 hydrogels: shear strain scanning curves (f), viscosity‐shear rate curve (g), and shear strain cycling measurement (h). i, j) Photographs of DN‐SPD 100 hydrogels formed in situ on pig skin subjected to various forces. Data are presented as mean ± SD in (b) and (d).

Porous structure is a crucial feature for ideal wound dressings.^[^ ^18^, ^21^ ^]^ Scanning electron microscopy (SEM) images of DN and DN‐SPD 100 hydrogels revealed uniform and interconnected 3D porous structures (**Figure** **1**a). Compared to DN hydrogel, the introduction of SPD into the DN‐SPD 100 hydrogel results in a denser microstructure with smaller pores. It is known that the crosslinking density plays a major role in determining the pore size of hydrogels.^[^ ^22^ ^]^ The addition of SPD can increase the number of amino groups to be crosslinked by the aldehyde groups of 4aPEG‐BA, yielding higher crosslinking density and smaller pores. This result is corroborated by the swelling ratios of the resulting hydrogels, which decrease as the concentration of SPD increases (Figure 1b). Specifically, the swelling ratios of DN, DN‐SPD 50, and DN‐SPD 100 hydrogels are 29.41 ± 0.93, 28.92 ± 0.74, and 24.07 ± 1.03, respectively. Notably, DN‐SPD 100 and DN‐DETA 100 hydrogels show comparable swelling ratios, suggesting that these two hydrogels are physically similar. More importantly, these hydrogels still maintain a certain level of mechanical strength at swelling equilibrium and can be handled by tweezers without damage. Indeed, the DN‐SPD hydrogel before swelling can withstand severe stretching, bending, or twisting without breakage, indicating excellent toughness and elasticity (Figure 1c). The compression moduli of the hydrogels were measured (Figure 1d; Figure S2, Supporting Information). As expected, the photo‐crosslinked DN hydrogel exhibits a higher compression modulus than the SN hydrogel; and the introduction of either SPD or DETA can further improve the compressive strength. In particular, DN‐SPD 100 is able to withstand a stress of more than 200 kPa at 70% strain and gives a compression modulus as high as 23 kPa, which is close to that of natural soft tissue.^[^ ^23^ ^]^

The resulting SN hydrogels obtained with SPD (SN‐SPD 100 and SN‐SPD 250) show good injectability and self‐healing properties, owing to the dynamic nature of imine bonds.^[^ ^24^ ^]^ They can be continuously extruded from a syringe without blocking the needle and regain the gel state immediately after injection (Figure 1e; Figure S3 and Video S1, Supporting Information). In addition, when SN‐SPD 100 and SN‐SPD 250 hydrogels of two different colors were cut and swapped, they could self‐heal within ≈10 min at 37 °C without any external force (Figure 1e; Figure S3, and Videos S2 and S3, Supporting Information). Moreover, SN‐SPD hydrogels can also conform to a heart‐shaped mold (Figure 1e), which is desirable for healing an irregular wound.

The injectability of the SN‐SPD 100 hydrogel was further characterized by rheological analysis (Figure 1f–g). In the shear strain range of 0.1% – 100%, the storage modulus (G′) is higher than the loss modulus (G″), indicating a stable gel state (Figure 1f). However, when the shear strain exceeds 100%, G′ steeply declines to be lower than G″, suggesting that the internal network of the hydrogel is damaged due to high shear stress. Cohesively, the viscosity of SN‐SPD 100 hydrogel decreases as the shear rate increases from 0.1 to 100 s^−1^ (Figure 1g), indicating a shear‐thinning feature that is preferred by 3D bio‐printing.^[^ ^25^ ^]^ The self‐healing performance of SN‐SPD 100 hydrogel was evaluated by cycling between 1% and 500% shear strain (Figure 1h). At 1% shear strain, G′ is higher than G″, whereas at 500% shear strain, G′ is lower than G″. Both G′ and G″ show quick response to the change of shear strain and rapidly return to previous values when the shear strain is back to 1%. After 3 cycles, G′ and G″ still display fast recovery, indicating an excellent self‐healing property for SN‐SPD 100 hydrogel.

Tissue adhesion is also important in a wound dressing.^[^ ^26^ ^]^ To this end, an SN‐SPD 100 hydrogel was printed and photo‐crosslinked in situ on pig skin. The in situ formed DN‐SPD 100 hydrogel can not only endure repeated bending and twisting but also carry a weight of up to 150 g (Figure 1i,j), indicating a strong tissue adhesion capability. In comparison, SN‐SPD 100 hydrogel formed on the surface of pig skin without photo‐crosslinking can only withstand simple bending and twisting, and carry a weight of ≈20 g, exhibiting a much less adhesion strength (Figure S4, Supporting Information). Hence, the tissue adhesion of our hydrogel can be significantly improved after in situ photo‐crosslinking.

### 2.2 Biological Effects of DN‐SPD Hydrogel In Vitro

**Figure 2:**
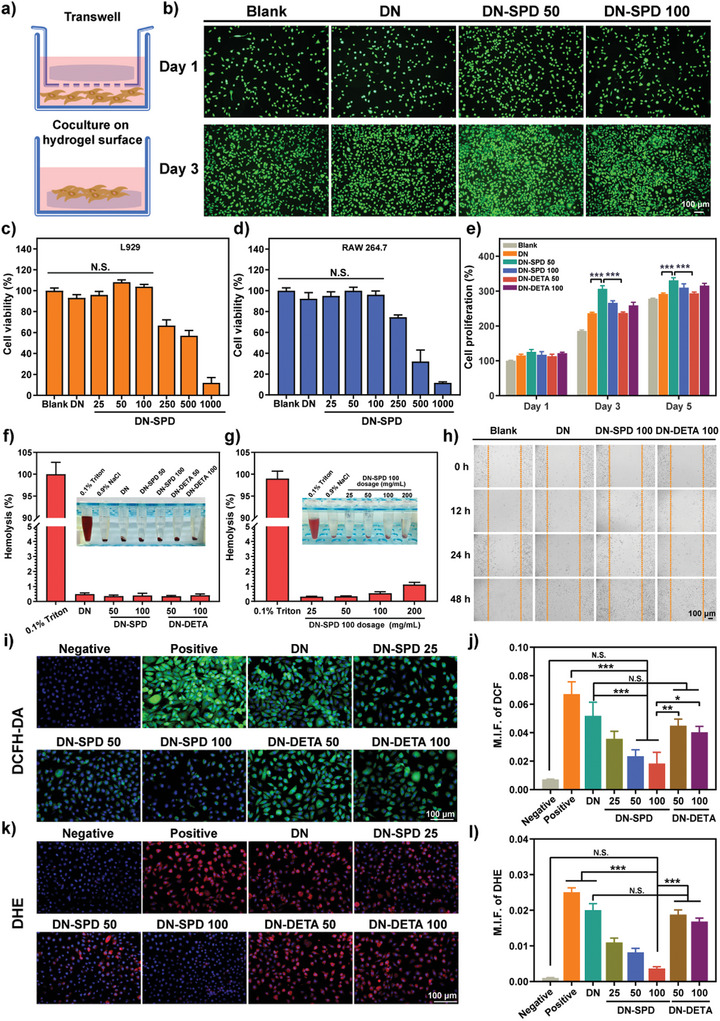
Biological effects of hydrogels in vitro. a) Diagram of two coculturing systems with hydrogels. b) Images of Live/Dead assay on L929 cells cocultured in transwell for 1 and 3 days. c, d) Cell viabilities of L929 (c) and RAW 264.7 (d) cells cocultured with various hydrogels in transwell for 24 h (*n* = 4). e) Proliferation of L929 cells cocultured on various hydrogels for 1, 3, and 5 days (*n* = 4). f, g) Hemolysis for various hydrogels (f) and different dosages of DN‐SPD 100 hydrogel (g) (*n* = 3). h) Photographs of scratch healing assay. i, j) Fluorescent images of DCFH‐DA probing for total intracellular ROS (i) and mean fluorescence intensity (M.F.I.) of DCF (j) (*n* = 3). k, l) Fluorescent images of DHE probing for intracellular superoxide anion (k) and M.F.I. of DHE (l) (*n* = 3). Data are presented as mean ± SD in (c‐g), (j), and (l). Statistical significance was determined by using one‐way ANOVA with Tukey's multiple comparisons (GraphPad Prism 8.0). ^*^: *p *< 0.05; ^**^: *p *< 0.01; ^***^: *p *< 0.001; N.S.: not significant.

Two methods were adopted to evaluate the cytocompatibility of different hydrogels: coculturing in a transwell and coculturing on the surface of a hydrogel (**Figure** **2**a). The Live/Dead assay on L929 cells cocultured with hydrogels in transwells shows that DN‐SPD 50 and DN‐SPD 100 hydrogels induce no observable cell death (Figure 2b). Similarly, the results of CCK‐8 assays of L929 and RAW 264.7 cells cultured on DN‐SPD hydrogels of various SPD concentrations also indicate a good cytocompatibility up to 100 µM of SPD (Figure 2c,d). Furthermore, after 3 and 5 days of coculturing, cell proliferation on hydrogels outperformed that of control without hydrogels (Figure 2e), suggesting that the hydrogels prepared in this work could favor cell proliferation.

Hemocompatibility was assessed by hemolytic rate (HR) for the various hydrogels. The HRs of DN, DN‐SPD 50/100, and DN‐DETA 50/100 are below 1% (Figure 2f), indicating excellent compatibility with blood cells. Moreover, the HR for DN‐SPD 100 hydrogel is only 1.12% at the dosage of 200 mg mL^−1^ (Figure 2g), well below the ASTM standard (<5%; F756:2008).^[^ ^27^ ^]^ Subsequently, the effects of hydrogels on cell migration were examined by scratch healing assay. Compared with the blank control, the hydrogel groups can promote the migration of L929 cells (Figure 2h). After 48 h, the scratch has been almost completely healed for the hydrogel groups. It is noted that DN‐SPD 100 and DN‐DETA 100 hydrogels yield greater cell density in the scratched area than DN hydrogel.

The complex microenvironment of diabetic wounds is usually accompanied by excessive production of reactive oxygen species (ROS).^[^ ^28^ ^]^ Encouraged by the ROS scavenging effects of the hydrogels in chemical experiments (Figure S5, Supporting Information), antioxidant activity at the cellular level was examined. Here, cellular oxidative stress was induced by exposing L929 cells to 100 mM H_2_O_2_. 2’,7’‐dichlorofluorescin diacetate (DCFH‐DA) was used to detect total ROS, whereas dihydroethidium (DHE) was utilized to probe superoxide anions (•O_2_^−^). Strong green fluorescence of 2’,7’‐dichlorofluorescin (DCF) is observed for the positive control, which can be reduced by DN‐SPD hydrogels in a dose‐dependent manner (Figure 2i,j). Consistently, the red fluorescence of DHE also displays a declining trend as the concentration of SPD increases (Figure 2k,l). Indeed, the fluorescence signals of DCF and DHE for DN‐SPD 100 hydrogel are similar to those of negative control without H_2_O_2_ treatment, implicating an outstanding antioxidant property. Intriguingly, DN‐DETA hydrogels yield less antioxidant activity than DN‐SPD hydrogels in cell experiments, whereas the antioxidant activities of DN‐SPD and DN‐DETA hydrogels are comparable in chemistry experiments (Figure S5, Supporting Information). Such an observation is in line with the report that SPD can reduce oxidative damage in old mice.^[^ ^29^ ^]^ In our case, both DN‐SPD and DN‐DETA hydrogels can provide chemical activity to scavenge ROS in acellular experiments, but the add‐up of the biological function of SPD makes DN‐SPD hydrogel better at antioxidation than DN‐DETA hydrogel in cell experiments.

### 2.3 Immunoregulation by DN‐SPD Hydrogel In Vitro

**Figure 3:**
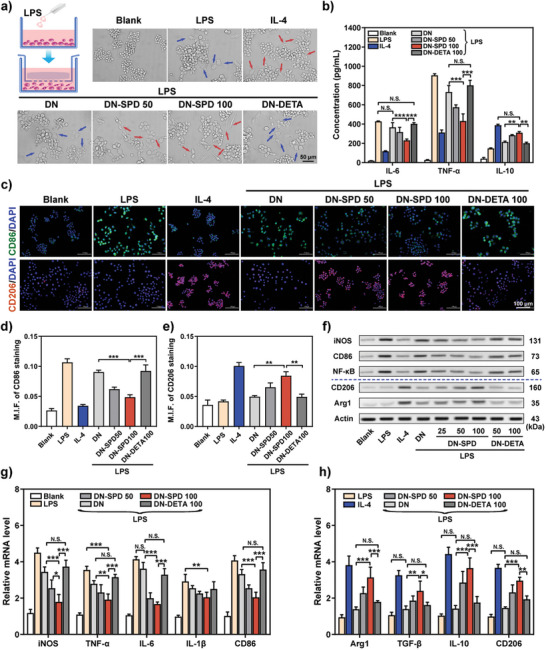
Immunoregulation by DN‐SPD hydrogel in vitro. a) Diagram of RAW 264.7 macrophages cocultured with hydrogels for LPS stimulation. b) The productions of IL‐6, TNF‐α, and IL‐10 for LPS‐stimulated RAW 264.7 cells cocultured with hydrogels. c) Fluorescent images of CD86 (green) and CD206 (red) staining at 48 h. d, e) M.F.I. of CD86 (d) and CD206 (e). f) Western blots for LPS‐stimulated RAW 264.7 cells cocultured with different hydrogels for 48 h. g, h) Relative mRNA levels of M1 macrophage markers (g) and M2 macrophage markers (h). Data are presented as mean ± SD (*n* = 3) in (b), (d), (e), (g), and (h). Statistical significance was determined by using one‐way ANOVA with Tukey's multiple comparisons (GraphPad Prism 8.0). ^*^: *p *< 0.05; ^**^: *p *< 0.01; ^***^: *p *< 0.001; N.S.: not significant.

Macrophages are key players in host defense and wound healing.^[^ ^30^ ^]^ Two major types of macrophages are M1 phenotype (classically activated macrophages), which promotes inflammatory responses, and M2 phenotype (alternatively activated macrophages), which resolves inflammation and repairs tissue.^[^ ^13^, ^31^ ^]^ Previously, we have demonstrated that SPD‐functionalized biomaterials can regulate macrophage polarization.^[^ ^18^ ^]^ Herein, more thorough investigations have been conducted with DN‐SPD hydrogels. It is found that RAW 264.7 macrophages cocultured with DN‐SPD hydrogels display elongated spindle‐like shape with long pseudopodia (**Figure** **3**a), which is characteristic of M2 phenotype and resembles the cell morphology of the positive control treated with interleukin‐4 (IL‐4). In contrast, the macrophages with DN and DN‐DETA hydrogels exhibit the shape of “fried eggs” with multiple pseudopodia, which is in line with the negative control stimulated with lipopolysaccharide (LPS) and thus represents M1 phenotype (Figure 3a). Cohesively, the LPS‐stimulated production of pro‐inflammatory cytokines, such as interleukin‐6 (IL‐6) and tumor necrosis factor‐α (TNF‐α), are significantly reduced by DN‐SPD hydrogels (Figure 3b). DN and DN‐DETA hydrogels yield no significant reduction on IL‐6 level and a much less decrease of TNF‐α production. Meanwhile, the level of anti‐inflammation cytokine, interleukin 10 (IL‐10), has been increased by DN‐SPD 100 hydrogel to a level close to that of the positive control (IL‐4), whereas the effects of DN and DN‐DETA 100 hydrogels on IL‐10 are minimal (Figure 3b).

Immunofluorescence of M1 and M2 macrophage markers, CD86 and CD206 respectively, reveals that the expression of CD86 (green) can be significantly suppressed by DN‐SPD hydrogel, but not by DN and DN‐DETA hydrogels. Instead, more positive staining of CD206 (red) can be observed for DN‐SPD hydrogel (Figure 3c–e). The inhibition by DN‐SPD hydrogel on M1 macrophage markers, e.g. inducible nitric oxide synthase (iNOS), CD86, and nuclear factor kappa‐B (NF‐κB), has been confirmed by Western blotting at protein level (Figure 3f), where the enhanced expression of M2 macrophage markers by DN‐SPD hydrogel, i.e. CD206 and argininase‐1 (Arg1), has also been verified. Meanwhile, we have examined the mRNA levels of various M1 phenotype markers, iNOS, TNF‐α, IL‐6, interleukin 1β (IL‐1β) and CD86, and M2 phenotype markers, Arg1, transforming growth factor‐β (TGF‐β), IL‐10 and CD206 by RT‐qPCR (Figure 3g,h). Consistently, the mRNA levels of M2 macrophage markers have been significantly elevated by DN‐SPD hydrogel in contrast to DN and DN‐DETA hydrogels, while the mRNA levels of M1 macrophage markers show opposite results. It should also be noted that the changes of the genes discussed herein, at both protein and mRNA levels, depend on the concentration of SPD, as DN‐SPD 100 hydrogel yields larger variation than DN‐SPD 50 hydrogel (Figure 3b–h). Taken together, these results suggest that DN‐SPD hydrogel can effectively promote the conversion of pro‐inflammatory M1 macrophages to anti‐inflammatory M2 macrophages, corroborating our previous findings.^[^ ^18^ ^]^

### 2.4 Reduction of Inflammatory Responses by DN‐SPD Hydrogel In Vivo

**Figure 4:**
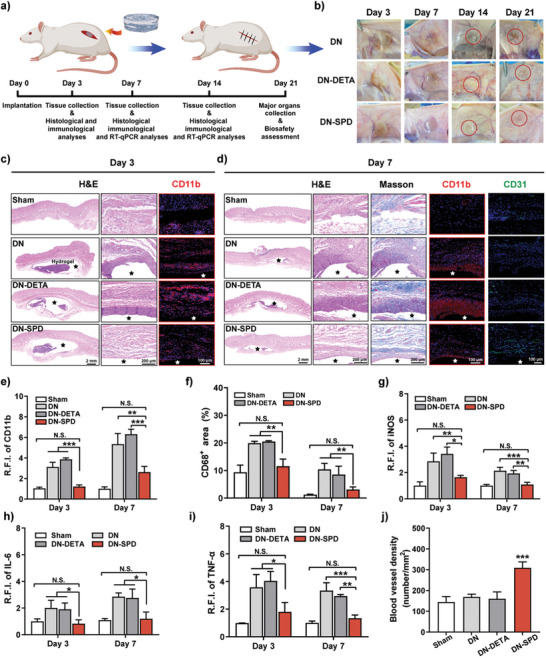
Immunoregulation by DN‐SPD hydrogel in vivo. a) Schematic illustration of subcutaneous implantation in rats. b) Images of implanted hydrogels on postsurgical Day 3, 7, 14 and 21. c) H&E and CD11b staining images of hydrogels on Day 3. d) H&E, Masson's, CD11b and CD31 staining images of hydrogels on Day 7. e, g‐i) Quantified relative fluorescence intensities (R.F.I.) of CD11b (e), iNOS (g), IL‐6 (h), and TNF‐α (i) (*n* = 4). f) Quantified CD68^+^ area (*n* = 4). j) Blood vessel density quantified by CD31 staining on Day 7 (*n* = 3). Data are presented as mean ± SD in (e‐j). Statistical significance was determined by using one‐way ANOVA with Tukey's multiple comparisons (GraphPad Prism 8.0). ^*^: *P *< 0.05; ^**^: *p *< 0.01; ^***^: *p *< 0.001; N.S.: not significant.

DN, DN‐SPD 250 (DN‐SPD hereafter), and DN‐DETA 250 (DN‐DETA hereafter) hydrogels were implanted subcutaneously and sutured in rats to investigate the immunoregulatory effects in vivo. (**Figure** **4**a). Macroscopic observation of the hydrogels and surrounding tissues on Day 3 and Day 7 after implantation shows that DN‐SPD hydrogel is devoid of tissue deposition and thus more transparent. On the contrary, DN and DN‐DETA hydrogels are both blurry with tissue deposition, and their surrounding tissues are swollen and inflamed (Figure 4b). H&E staining was conducted to examine the immune response at the interface of the implanted hydrogels and host tissues. On postsurgical Day 3 and Day 7, thick layers of immune cells have accumulated around DN and DN‐DETA hydrogels (Figure 4c,d), whereas only a thin layer of immune cells is surrounding DN‐SPD hydrogel indicating a significantly lower inflammatory response. On Day 14, the inflammation around DN and DN‐DETA hydrogels is still obvious, but the inflammation around DN‐SPD hydrogel has been almost resolved (Figure S6a, Supporting Information). These findings have been corroborated by immunofluorescent staining against CD11b (Figure 4c–e), a common marker of immune cells. More importantly, immunofluorescent staining against CD68 (Figure 4f; Figure S7, Supporting Information), a pan‐macrophage marker, shows that the density of macrophages in tissues surrounding DN‐SPD hydrogel is comparable to that of sham operation and remarkably lower than those of DN and DN‐DETA hydrogels. Further characterization of macrophage phenotype by M1 macrophage marker (iNOS) and M2 macrophage markers (CD163 and Arg1) reveals a lower proportion of pro‐inflammatory M1 phenotype and a higher proportion of anti‐inflammatory M2 phenotype for DN‐SPD hydrogel (Figure 4g, Figures S7 and S8, Supporting Information). Corroborative, immunofluorescent staining of IL‐6 and TNF‐α also indicates significantly reduced inflammatory responses for DN‐SPD hydrogel in comparison with DN and DN‐DETA hydrogels (Figure 4h,i; Figure S8, Supporting Information).

Next, Masson's trichrome staining was used to evaluate collagen deposition around implanted hydrogels, which could serve as an indicator of fibrous encapsulation around implants and thus foreign body response (FBR).^[^ ^32^ ^]^ DN‐SPD hydrogel incurred much less collagen deposition than DN and DN‐DETA hydrogels on Day 7 (Figure 4d). After two weeks of implantation, only sparse collagen fibers can be observed for DN‐SPD hydrogel, while DN and DN‐DETA hydrogels are surrounded by dense collagen fibers (Figure S6a, Supporting Information). The quantified collagen density around DN‐SPD hydrogel is 46.54% ± 5.34%, which is much lower than the collagen densities around DN (74.41% ± 4.18%) and DN‐DETA (82.69% ± 5.34%) hydrogels (Figure S6b, Supporting Information), implying an evidently decreased fibrous encapsulation around DN‐SPD hydrogel. Furthermore, immunofluorescent staining against CD31 indicates better neovascularization in the tissue surrounding DN‐SPD hydrogel on Day 7 (Figure 4d,j).

Moreover, RT‐qPCR was employed to verify the above results at mRNA level. Consistently, the mRNA levels of CD11b, CD68, IL‐6, and TNF‐α as well as CD86 (M1 macrophage marker) are significantly reduced for DN‐SPD hydrogel on Day 7, compared to those of DN and DN‐DETA hydrogels, while the mRNA levels of CD206 (M2 macrophage marker) and CD31 are dramatically increased (Figure S9, Supporting Information).

**Figure 5:**
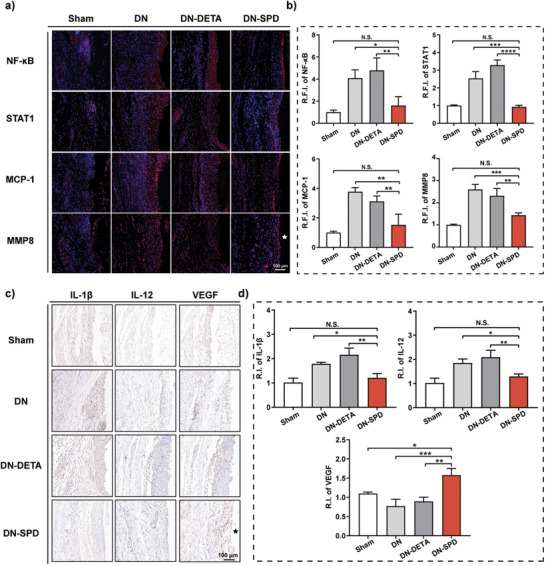
Expressions of inflammation mediators and VEGF. a) Immunofluorescent staining of inflammation mediators (NF‐κB, STAT1, MCP‐1, and MMP8) in tissues surrounding different hydrogels. b) Quantified R.F.I. of inflammation mediators. c) Immunohistochemical staining of IL‐1β, IL‐12, and VEGF in tissues surrounding different hydrogels. d) Quantified relative intensities (R.I.) of IL‐1β, IL‐12, and VEGF. Data are presented as mean ± SD (*n* = 4) in (b) and (d). Statistical significance was determined by using one‐way ANOVA with Tukey's multiple comparisons (GraphPad Prism 8.0). ^*^: *p *< 0.05; ^**^: *p *< 0.01; ^***^: *p *< 0.001; N.S.: not significant.

In our previous work, several inflammation mediators have been found down‐regulated by the comparison of SPD‐functionalized biomaterials vs. DETA‐functionalized biomaterials, including NF‐κB, signal transducers and activators of transcription 1 (STAT1), monocyte chemoattractant protein‐1 (MCP‐1) and matrix metalloproteinase 8 (MMP8).^[^ ^18^ ^]^ Herein, we have confirmed the down‐regulation of these proteins in the tissues around DN‐SPD hydrogel by immunofluorescence on Day 7 (**Figure** **5**a,b). Cohesively, the expressions of two more pro‐inflammatory cytokines, IL‐1β and interleukin 12 (IL‐12), are also found to be reduced for DN‐SPD hydrogel by immunohistochemistry and RT‐qPCR (Figure 5c,d; Figure S9, Supporting Information). Indeed, the expressions of these inflammation mediators in tissues around DN‐SPD hydrogel are even close to the levels of sham operation, in dramatic contrast to those of DN and DN‐DETA hydrogels. In addition, immunohistochemical staining for vascular endothelial growth factor (VEGF), a common angiogenic factor, shows up‐regulated VEGF expression at the implantation site (Figure 5c,d), which is in line with the immunofluorescence result of CD31 (Figure 4d,j). The up‐regulation of VEGF has also been confirmed by RT‐qPCR at mRNA level (Figure S9j, Supporting Information).

Together, these results indicate that subcutaneous implantation of DN and DN‐DETA hydrogels could trigger a serious inflammatory response and fibrous encapsulation in rats as a consequence of FBR, which can be alleviated by DN‐SPD hydrogel. In addition, our findings also suggest that SPD can reduce the implant‐induced inflammatory responses by altering the polarization of macrophages, given that DN and DN‐DETA hydrogels show no significant difference, while evident anti‐inflammatory effects have been observed only for DN‐SPD hydrogel.

It should also be noted that DN, DN‐SPD, and DN‐DETA hydrogels are almost completely degraded in vivo after 21 days of implantation (data not shown), which is faster than their in vitro degradation (Figure S10, Supporting Information). For in vitro degradation, DN‐SPD 100 and DN‐DETA 100 hydrogels demonstrate similar degradation behavior and roughly 63.1% of the hydrogels remain after 21 days in phosphate‐buffered saline (PBS). Meanwhile, DN and DN‐SPD 50 hydrogels exhibit faster in vitro degradation with 47.8% and 55.4% retained after 21 days (FigureS10, Supporting Information), which indicates that the loading of SPD favors longer degradation. Moreover, no pathological changes in major organs have been found for these hydrogels after 21 days of implantation, including the heart, liver, spleen, lung, and kidney (Figure S11, Supporting Information). Hence, these hydrogels have demonstrated good biodegradability and safety.

### 2.5 Enhanced Acute Wound Healing by DN‐SPD Hydrogel in Normal Mice

**Figure 6:**
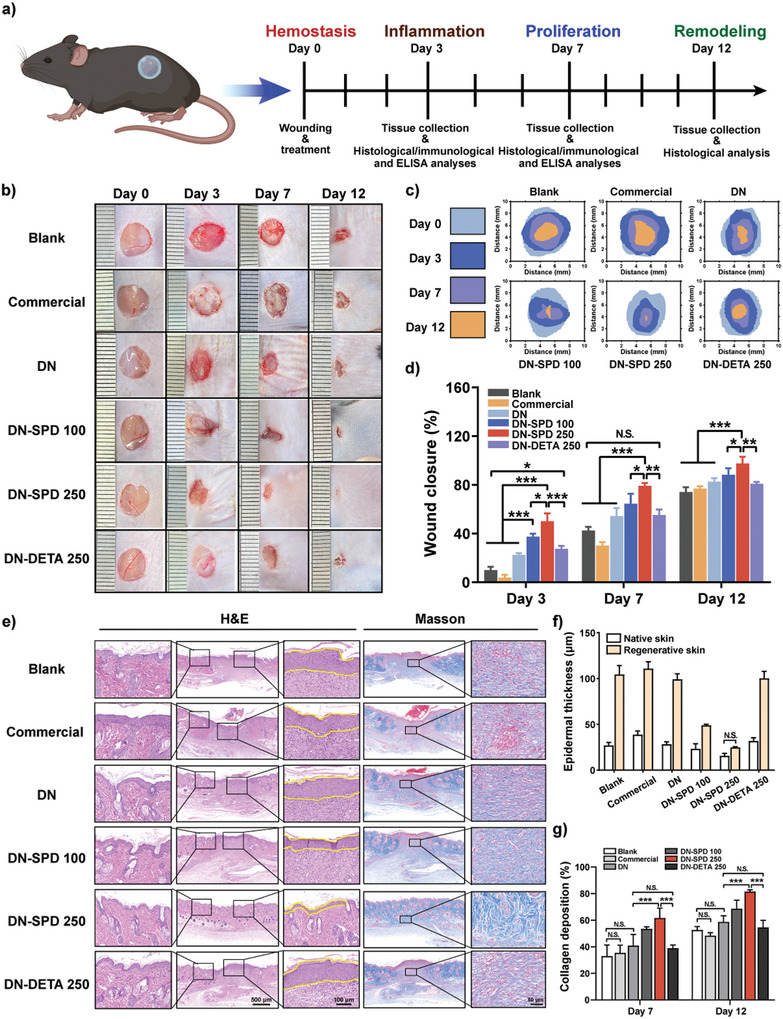
Enhanced acute wound healing by DN‐SPD hydrogel in normal mice. a) Schematic illustration of the experiments in normal mice. b) Images of the wounds on Day 0, 3, 7 and 12. c) Wound closure traces during the healing. d) Quantified wound closure rates (*n* ≥ 3). e) H&E and Masson's trichrome staining of wounds on Day 12. f) Quantified epidermal thickness on Day 12 (*n* = 3). g) Quantified collagen deposition (*n* = 3). Data are presented as mean ± SD in (d), (f), and (g). Statistical significance was determined by using one‐way ANOVA with Tukey's multiple comparisons (GraphPad Prism 8.0). ^*^: *p *< 0.05; ^**^: *p *< 0.01; ^***^: *p *< 0.001; N.S.: not significant.

Full‐thickness skin defect model (8 mm in diameter) was established in C57BL6 mice to investigate the effects of SPD‐functionalized hydrogels on acute wound healing (**Figure** **6**a). The wounds were treated with DN, DN‐SPD 100, DN‐SPD 250, and DN‐DETA 250 hydrogels, together with PBS (Blank) and Duo DERM (Commercial), and photographed on Day 0, 3, 7, and 12 (Figure 6b,c). The wounds treated with DN‐SPD 100 and DN‐SPD 250 hydrogels show significantly faster healing than the other groups. On Day 7, the wound closure rates of DN‐SPD 100 and DN‐SPD 250 hydrogels are 64.5% and 79.2%, whereas the closure rates of Blank, Commercial, DN and DN‐DETA 250 are 30.0%, 42.5%, 50.4% and 55.1%, respectively (Figure 6d). Such an effect is believed to depend on SPD for two reasons. First, the healing by DN‐DETA 250 is comparable to that of DN, whereas accelerated healing is observed for DN‐SPD 100 and DN‐SPD 250 hydrogels. Second, a higher concentration of SPD yields faster healing (DN vs. DN‐SPD 100 vs. DN‐SPD 250), suggesting a dose dependence on SPD. On Day 12, the wounds of DN‐SPD 250 group have been almost completely healed (closure rate of 95.5%), while unclosed wounds are still visible in the other groups.

H&E staining was performed to evaluate the regenerated skins histologically. The infiltration of immune cells has been significantly reduced in the DN‐SPD 100 and DN‐SPD 250 groups on Day 3 and 7 (Figure S12, Supporting Information). On Day 7, the granular tissue began to form in the DN‐SPD 100 and DN‐SPD 250 groups, and the wound treated with DN‐SPD 250 hydrogel was smaller than the other groups (Figure S12b, Supporting Information). On Day 12, a thinner newly formed epidermis is found for the DN‐SPD 100 and DN‐SPD 250 groups, while the epidermal layers in the other groups are much thicker (Figure 6e,f). In particular, the epidermal thickness of DN‐SPD 250 group is comparable to that of native skin (Figure 6f). Collagen analysis by Masson's trichrome staining reveals that DN‐SPD 250 hydrogel promoted more collagen deposition on Day 7 (Figure 6g; Figure S12b, Supporting Information) and yielded more mature collagen on Day 12 (Figure 6e). Specifically, the collagen fibers in DN‐SPD 250 group are more regularly arranged in a pattern like a woven basket, which resembles the native skin (Figure 6e; Figure S13, Supporting Information). In contrast, the other groups exhibit not only less collagen deposition but also more randomly oriented collagen fibers (Figure 6e,g; Figure S12b, Supporting Information). Furthermore, the immunohistochemical staining of collagen I and III has also confirmed that more of both are deposited in the DN‐SPD 250 group (Figure S14, Supporting Information). Angiogenesis is another important indicator of wound healing. Immunofluorescent staining of CD31, a marker of newly formed blood vessels, shows higher blood vessel density for the DN‐SPD 250 group than the other groups on Day 7 (Figure S15, Supporting Information), in consistency with the results of subcutaneous implantation in rats (Figure 4d,j). Interestingly, a lower level of CD31 is found for the DN‐SPD 250 group on Day 12, indicating less neovascularization at this stage. Nevertheless, the staining against α‐smooth muscle actin (α‐SMA), a vascular smooth muscle cell marker, reveals more vascular structures than the other groups (Figure S15a, Supporting Information). These findings are in line with our previous results^[^ ^18^ ^]^ and likely because the wound treated by DN‐SPD 250 hydrogel has regenerated enough blood vessels on Day 12 and thus requires less neovascularization, whereas abundant new blood vessels are still being regenerated for the other groups. The above results suggest that DN‐SPD 250 hydrogel can achieve faster and more natural wound healing, accompanied by better collagen deposition and angiogenesis.

**Figure 7:**
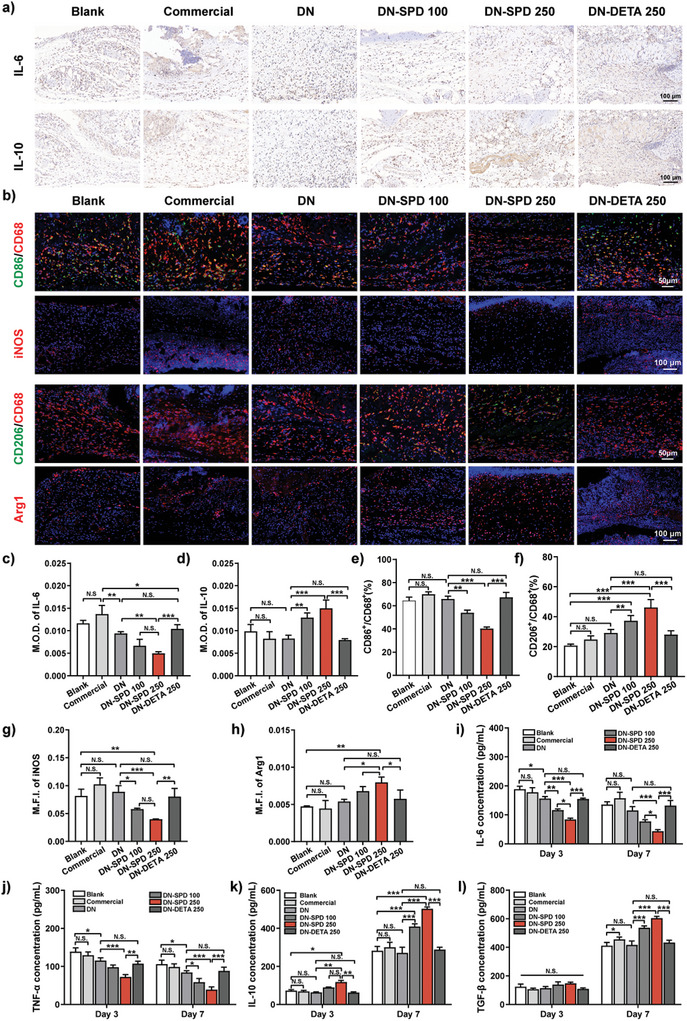
Immunoregulation by DN‐SPD hydrogel in acute wound healing. a) Immunohistochemical staining of IL‐6 and IL‐10 on Day 3. b) Immunofluorescent staining of CD86/CD68, iNOS, CD206/CD68, and Arg1 on Day 3. c, d) Mean optical densities (M.O.D.) for IL‐6 (c) and IL‐10 (d) (*n* = 4). e, f) Quantified ratios of CD86^+^/CD68^+^ (e) and CD206^+^/CD68^+^ (f) (*n* = 4). g, h) Quantified M.F.I. for iNOS (g) and Arg1 (h) (*n* = 4). i‐k) ELISA measurements of IL‐6 (i), TNF‐α (j), IL‐10 (k), and TGF‐β (l) in the wound tissue lysates extracted on days 3 and 7 (*n* = 3). Data are presented as mean ± SD in (**c‐l**). Statistical significance was determined by using one‐way ANOVA with Tukey's multiple comparisons (GraphPad Prism 8.0). ^*^: *p *< 0.05; ^**^: *p *< 0.01; ^***^: *p *< 0.001; N.S.: not significant.

A possible reason for delayed wound healing is sustained inflammation. To this end, we have quantified the levels of representative cytokines and markers in wound tissues by immunohistochemistry and immunofluorescence (**Figure** **7**). High levels of pro‐inflammatory IL‐6 and low levels of anti‐inflammatory IL‐10 are found for the Blank, Commercial, and DN groups on Day 3 (Figure 7a,c,d) and 7 (Figure S16, Supporting Information). Meanwhile, the DN‐DETA 250 group shows results comparable to those of DN group, indicating no immunomodulatory effect for DETA. In contrast, for the DN‐SPD 100 and DN‐SPD 250 groups, the level of IL‐6 is reduced and the level of IL‐10 is increased in a dose‐dependent manner, further confirming the immunoregulatory role of SPD. Consistently, the characterization of M1 macrophage by CD68, CD86, and iNOS staining reveals a lower CD86^+^/CD68^+^ ratio and reduced level of iNOS for the DN‐SPD 250 group during the inflammatory phase (Day 3) (Figure 7b,e,g). On the contrary, the staining of M2 phenotype markers, CD206 and Arg1, displays a higher CD206^+^/CD68^+^ ratio and Arg1 level for the DN‐SPD 250 group during both the inflammatory phase (Day 3) and proliferation phase (Day 7) (Figure 7b,f,h; Figure S17, Supporting Information). Again, no significant alteration of macrophage phenotype is found in the comparison of the DN‐DETA 250 and DN groups, and fewer changes can be observed for the DN‐SPD 100 group. Furthermore, measurements of key inflammation‐related cytokines by enzyme‐linked immunosorbent assay (ELISA) exhibit decreased levels of pro‐inflammatory IL‐6 and TNF‐α for the DN‐SPD 250 group on Day 3 and 7 as well as enhanced levels of anti‐inflammatory IL‐10 and TGF‐β (Figure 7i–l). Hence, our results suggest that the faster wound healing observed for DN‐SPD 250 hydrogel is endowed by the immunoregulatory role of SPD, which accelerates the transformation of M1 macrophages to M2 macrophages and resolution of inflammation while down‐regulating pro‐inflammatory cytokines and up‐regulating anti‐inflammatory cytokines.

### 2.6 Enhanced Diabetic Wound Healing by In Situ DN‐SPD Hydrogel

**Figure 8:**
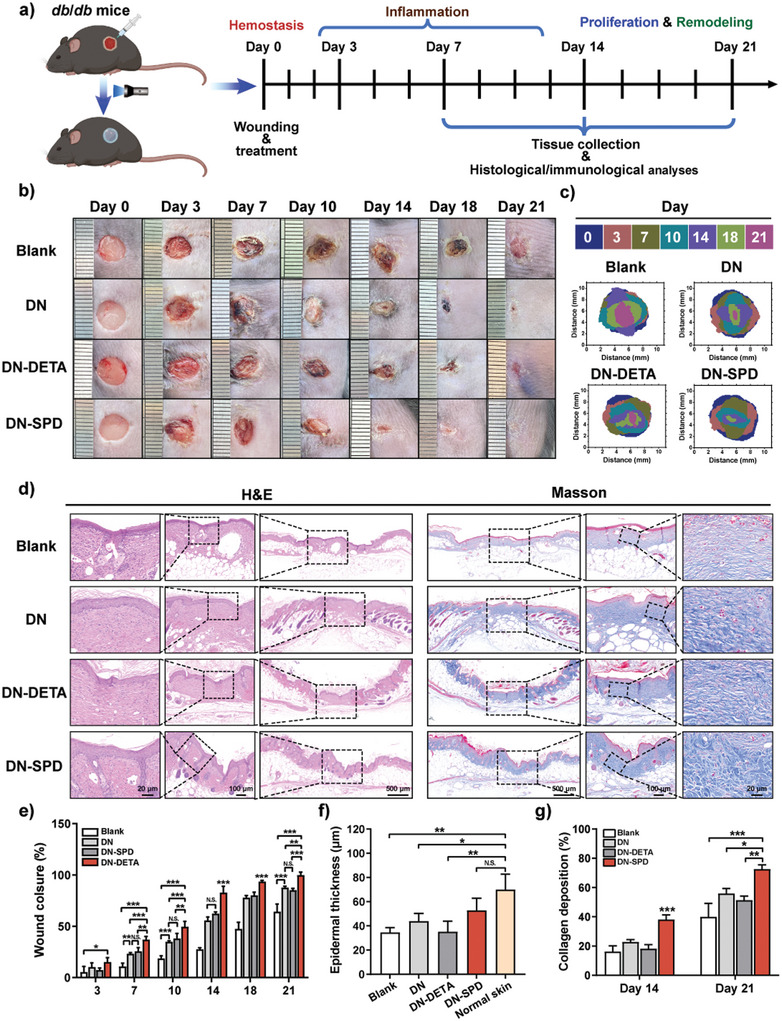
Enhanced diabetic wound healing by in situ formed DN‐SPD hydrogel. a) Schematic illustration of the experiments in *db*/*db* mice with DN, DN‐SPD 250, and DN‐DETA 250 hydrogels. b) Images of the wounds on Day 0, 3, 7, 10, 14, 18 and 21. c) Wound closure traces during the healing. d) H&E and Masson's trichrome staining of wounds on Day 21. e) Quantified wound closure rates (*n* ≥ 3). f) Quantified epidermal thickness on Day 21 (*n* = 3). g) Quantified collagen deposition (*n* = 3). Data are presented as mean ± SD in **e–g**). Statistical significance was determined by using one‐way ANOVA with Tukey's multiple comparisons (GraphPad Prism 8.0). ^*^: *p *< 0.05; ^**^: *p *< 0.01; ^***^: *p *< 0.001; N.S.: not significant.

Full‐thickness skin defect model (8 mm in diameter) was established in db/db mice to evaluate the therapeutic effect of DN‐SPD 250 hydrogel on more serious chronic refractory wounds (**Figure** **8**a). As aforementioned, SN‐SPD 250 hydrogel has good injectability and can be printed (Video S4, Supporting Information). Hence, unlike the previous procedure, DN, DN‐DETA 250 (hereafter DN‐DETA), and DN‐SPD 250 (hereafter DN‐SPD) hydrogels were printed onto the wound sites and irradiated with blue light to form hydrogels in situ in this experiment (Video S5, Supporting Information). Notably, the in situ formed hydrogels demonstrated robust adhesion to the wounds and were left without any other protection, corroborating with the results on pig skin (Figure 1i,j). The Blank group treated with PBS underwent a delayed healing process longer than 21 days, and purulent exudate caused by infection could be observed in the wound bed. (Figure 8b,c,e). Compared to the Blank group, hydrogel‐treated groups display faster wound healing, and again, the DN‐SPD group shows even faster healing than the DN and DN‐DETA groups. Specifically, the wound closure rate of DN‐SPD on Day 14 is 87.9%, significantly higher than those of DN (55.7%) and DN‐DETA (62.3%). On Day 18, 96.8% of the wound area was healed for DN‐SPD, whereas obvious wounds can still be seen for the other groups. More intriguingly, almost no pus has been observed for DN‐SPD during the healing.

The results of the histological analysis agree with the macroscopic observation. The DN‐SPD group exhibits smaller defects than the other groups on Day 7 and the difference between DN‐SPD and the other 3 groups is more evident on Day 14 (Figure S18, Supporting Information). More importantly, denser granular tissues can be observed for DN‐SPD on Day 14. On Day 21, the continuous epidermis formed on the hydrogel‐treated wounds, but not for the Blank group (Figure 8d). Yet, the newly formed skin of DN‐SPD is more similar to native skin in terms of epidermal thickness and hair follicles, while the epidermal thicknesses of the other groups are still significantly smaller (Figure 8f). The results of Masson's trichrome staining show an increase in collagen volume from Day 14 to Day 21 for all groups, among which the DN‐SPD group yields the highest collagen volume and most orderly arranged collagen, resembling normal skin (Figure 8d,g, and Figure S18b, Supporting Information). Furthermore, more matured collagens have been identified for the DN‐SPD group by Sirius red staining on Day 21 (Figure S19, Supporting Information), indicating better tissue remodeling. Hence, our results have demonstrated that in situ‐formed DN‐SPD hydrogel can effectively accelerate the prolonged healing of diabetic wounds, facilitating collagen deposition and maturation to restore the structure of normal skin.

**Figure 9:**
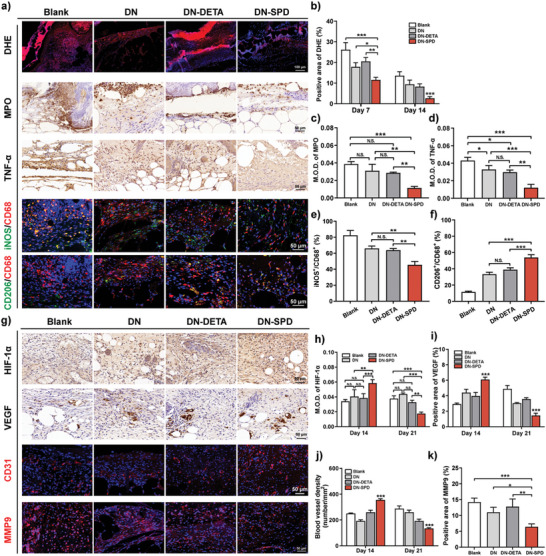
Immunoregulation by DN‐SPD hydrogel in diabetic wound healing. a) Immunofluorescent and immunohistochemical staining of DHE, MPO, TNF‐α, iNOS/CD68, and CD206/CD68 on Day 7. b) Quantified positive area of DHE. c, d) Quantified M.O.D. of MPO (c) and TNF‐α (d). e, f) Quantified ratios of iNOS^+^/CD68^+^ (e) and CD206^+^/CD68^+^ (f). g) Immunofluorescent and immunohistochemical staining of HIF‐1α, VEGF, CD31 and MMP9 on Day 14. h) Quantified M.O.D. of HIF‐1α. i, k) Quantified positive areas of VEGF (i) and MMP9 (k). j) Blood vessel density quantified by CD31 staining. Data are presented as mean ± SD (*n* = 3) in (b‐f) and (h‐k). Statistical significance was determined by using one‐way ANOVA with Tukey's multiple comparisons (GraphPad Prism 8.0). ^*^: *p *< 0.05; ^**^: *p *< 0.01; ^***^: *p *< 0.001; N.S.: not significant.

Prolonged inflammation and high levels of ROS are typical features of chronic refractory wounds.^[^ ^33^ ^]^ Particularly, it is known that local hyperglycemia in diabetic wounds can cause the accumulation of ROS.^[^ ^34^ ^]^ Therefore, DHE staining was conducted to examine the ROS levels in the wound tissues of *db*/*db* mice on Day 7 and 14 (**Figure** **9**a; Figure S20, Supporting Information). Consistent with our in vitro results (Figure 2i–l), the fluorescence intensity of DHE has been significantly reduced in the DN‐SPD group on Day 7 (Figure 9a,b), compared to the strong fluorescence observed for the Blank, DN, and DN‐DETA groups. After 14 days of treatment, almost no fluorescence of DHE can be found for DN‐SPD, whereas obvious red fluorescence can still be seen for the other groups. These results suggest that DN‐SPD hydrogel also possesses excellent antioxidant properties in vivo and can alleviate oxidative stress during wound healing.

To verify the prolonged inflammation phase of diabetic wound healing, the infiltration of neutrophils was characterized by immunohistochemical staining against myeloperoxidase (MPO), a marker of activated neutrophils.^[^ ^35^ ^]^ Abundant MPO‐positive neutrophils are identified for the Blank group on Day 7 (Figure 9a,c), confirming a persistent inflammation. In contrast, fewer MPO‐positive cells are found in hydrogel‐treated groups. Moreover, in comparison with the DN and DN‐DETA groups, even fewer MPO‐positive cells are observed for DN‐SPD. Cohesively, the immunohistochemical staining of TNF‐α also shows strong inflammation for Blank and significantly decreased inflammation for DN‐SPD on Day 7 (Figure 9a,d).

High levels of ROS can affect the transformation of macrophages, leading to the excessive accumulation of M1 macrophages.^[^ ^36^ ^]^ Double immunofluorescent staining of iNOS/CD68 for M1 macrophages and CD206/CD68 for M2 macrophages was carried out to investigate macrophage phenotypes in the diabetic wound tissues on Day 7. The DN‐SPD group exhibits a lower proportion of iNOS^+^ cells (M1 macrophages) and a higher proportion of CD206^+^ cells (M2 macrophages) than the other 3 groups (Figure 9a,e,f), corroborating with the aforementioned in vitro and in vivo results that DN‐SPD hydrogel favors M2 polarization of macrophages (Figure 3, Figure 4g, Figure 7b, e–h; Figures S7–S9, Supporting Information).

In addition, high levels of ROS and glucose in diabetic wounds are often associated with decreased levels of hypoxia‐induced factor‐1α (HIF‐1α), which in turn down‐regulates the expression of VEGF and thus impairs angiogenesis.^[^ ^37^ ^]^ The immunohistochemical staining for HIF‐1α reveals a higher level of HIF‐1α expression for DN‐SPD on Day 14 (Figure 9g,h) but lower expression of HIF‐1α on Day 21 (Figure 9h; Figure S21a, Supporting Information), in comparison with the other 3 groups. Therefore, immunohistochemical staining of VEGF and immunofluorescent staining of CD31 were performed to confirm the results of HIF‐1α staining. Again, significantly enhanced expressions of VEGF and CD31 are found for DN‐SPD on Day 14, whereas the expressions of these two markers of neovascularization are decreased on Day 21 (Figure 9g,i,j; Figures S21b and S22, Supporting Information). Similar trends have also been observed for CD31 in normal mice but within a shorter period, on Days 7 and 12 (Figure S15, Supporting Information). It is presumably because in the scenario of prolonged diabetic wound healing, insufficient blood vessels have been generated on Day 14, and the wound still suffers hypoxia and requires neovascularization. As a result, HIF‐1α, VEGF, and CD31 are up‐regulated by DN‐SPD hydrogel on Day 14. Yet on Day 21, the wound treated by DN‐SPD hydrogel has regenerated sufficient blood vessels to overcome hypoxia and thus demands less neovascularization, whereas the wounds of other groups are still under active neovascularization.

Matrix metalloproteinase 9 (MMP9) plays an important role in ECM degradation and tissue remodeling. It is known that the overexpression of MMP9 in diabetic wounds disrupts the balance between ECM synthesis and degradation, thus hampering the formation of granular tissue and delaying wound healing.^[^ ^38^ ^]^ Immunofluorescent staining of MMP9 exhibits less MMP9 for the DN‐SPD group than the other groups on Day 14 (Figure 9g,k), which is in line with the better skin regeneration and collagen deposition observed for DN‐SPD (Figure 8d,f,g). Together, we have shown that DN‐SPD hydrogel can reduce the prolonged inflammation and high ROS levels of diabetic wounds, which promotes M2 polarization of macrophages and improves angiogenesis in the early stage of wound healing. In addition, the reduced expression of MMP9 may afford another clue for faster and more natural skin regeneration by DN‐SPD hydrogel.

## 3 Discussion

SPD is an “old” molecule originally isolated from semen. Yet, not until this century have various “new” functions been found for SPD, such as autophagy induction,^[^ ^39^ ^]^ anti‐aging,^[^ ^40^ ^]^ lifespan extension,^[^ ^41^ ^]^ immunomodulation,^[^ ^9^ ^]^ metabolic regulation,^[^ ^42^ ^]^ neuroprotection,^[^ ^43^ ^]^ *etc*. The key functions of SPD have been comprehensively reviewed in an article published in *Science* in 2018.^[^ ^8a^ ^]^ However, the majority of SPD‐related research has been focused on the effects of systemically administrated SPD. In this work, we have demonstrated that the local administration of SPD in biomaterials can mitigate inflammation and FBR in a simple but more natural way, which opens up a door for medical implants based on exogenous biomaterials and even for transplanted organs.

First, a very critical control in our experiments should be emphasized, i.e. DETA. DETA closely resembles SPD in chemistry. Specifically, DETA is a symmetric molecule with two 2‐aminoethyl groups on each side of the secondary amine. In comparison with DETA, SPD has two more methylene groups on one side of the secondary amine and one more methylene group on the other side (Figure S1, Supporting Information), which makes SPD an asymmetric molecule with multiple roles. It is seemingly amazing that such a small difference in chemical structure can cause so distinct biological effects. Yet, we have to believe that Mother Nature is capable of finding the “right” molecule through evolution. An important implication of the simple chemical structure of SPD is that we don't need to isolate SPD from semen! Chemical synthesis of SPD can be easily done and thus the cost of SPD is low. Actually, the SPD used in our experiments was purchased from Sigma‐Aldrich.

Second, a higher concentration of SPD should be used in vivo than that of in vitro experiments. Based on our experience, free SPD compound shows no cytotoxicity on NIH/3T3 cells^[^ ^18^ ^]^ as well as L929 and RAW 264.7 cells (data not shown) up to 5 µM, but higher concentration of SPD greater than 10 µM may cause some cell death. It is also known that the concentration of SPD in normal human semen varies in the range of 0.25 to 1.17 mM,^[^ ^44^ ^]^ which is ≈2 magnitudes larger than the tolerable concentration in cell culture. This seeming discrepancy is due to the different scenarios of in vitro and in vivo experiments. For in vitro experiments, cells are cultured in a pond of culture medium without circulation. In contrast, in living animals or humans, circulating blood can effectively dilute and carry away a small molecule like SPD. In this work, we have found DN‐SPD 100 hydrogel exhibits better cell viability than DN‐SPD 250 hydrogel in vitro (Figure 2c,d), but DN‐SPD 250 hydrogel gives better healing outcome than DN‐SPD 100 hydrogel in vivo (Figure 6). Cohesively, in our previous work, we have also found that hydrogels and films with lower concentrations of SPD are better in cell culture experiments, whereas hydrogels and films loaded with higher concentrations of SPD perform better in animals.^[^ ^18^ ^]^

Third, SPD is a double‐side agent that knows how to achieve the natural outcome. Although SPD plays multiple roles, it is not a potent drug. It acts like traditional Chinese medicine, coordinating several biological pathways to yield a natural outcome synergistically. In this work, it has been shown that the subcutaneous implantation of SPD‐functionalized hydrogels induces less collagen deposition (Figure 4), whereas the acute skin wound healing with SPD‐functionalized hydrogels results in more collagen deposition on Day 7 and more mature collagen on Day 12 (Figure 6e,g; Figures S12b and S14, Supporting Information). In the scenario of subcutaneous implantation, the host recognizes the implanted materials as “foreign bodies” and triggers a complex signaling cascade of the immune system, which leads to severe inflammation, collagen deposition, and fibrous encapsulation on the implanted materials.^[^ ^32^, ^45^ ^]^ In addition, no new tissue generation is necessary at the implanted site since the linear incision has been sutured after implantation (Figure 4a). Hence, collagen deposition is regarded as a positive indicator of FBR after subcutaneous implantation. The administration of SPD by DN‐SPD 250 hydrogels at the implanted site reduces local inflammatory response as well as FBR leading to less collagen deposition and a more favorable outcome. In contrast, new tissue formation is needed for skin wound healing. As the main component of the extracellular matrix, collagen contributes to neovascularization and re‐epithelialization during wound healing.^[^ ^46^ ^]^ Therefore, proper collagen deposition and remodeling is one of the important indicators to monitor the healing process of skin wounds. In this case, the administration of SPD by DN‐SPD 250 hydrogels reduces inflammation and accelerates the transition to the proliferation phase of skin wound healing. Hence, more collagen deposition on Day 7 and more mature collagen on Day 12 have been observed (Figure 6e,g; Figures S12b and S14, Supporting Information), indicating faster and better wound healing. In summary, SPD‐functionalized hydrogels have yielded seemingly contradictory but more desirable results depending on the biological events.

Last but not least, it should be noted that the release behavior of SPD is still under investigation for our SPD‐functionalized hydrogels. Nevertheless, we intend to use the degradation of our hydrogels as an approximation for the release behavior of SPD. Hence, we have shown the degradation profiles of DN, DN‐SPD 50, DN‐SPD 100, and DN‐DETA 100 hydrogels in Figure S10 (Supporting Information). Meanwhile, based on our experience, the cell viabilities for DN‐SPD 100 and DN‐SPD 250 hydrogels (Figure 2c,d) correlate with those with 5 and 10 µM free SPD,^[^ ^18^ ^]^ respectively. In this sense, it is reasonable to assume that the majority of SPD is not released from the SPD‐functionalized hydrogels. Instead, the release of SPD from SPD‐functionalized hydrogels mainly depends on the degradation.

## 4 Conclusion

In summary, we have developed an injectable double network hydrogel functionalized with SPD for bio‐printing. The single network hydrogel with dynamic imine bonds but without photo‐crosslinking can be printed onto wounds and photo‐crosslinked in situ to form double network hydrogels. The double network hydrogels exhibit desirable mechanical properties and tissue adhesion. More importantly, an “operando” comparison of hydrogels loaded with SPD and DETA has shown similar physical properties, but quite different biological functions. In particular, the outcomes of 3 sets of in vivo animal experiments have collectively proved that the DN‐SPD hydrogels can not only reduce inflammation caused by implanted biomaterials and ROS, but also promote the polarization of macrophages toward regenerative M2 phenotype, whereas no such effects have been observed for the DN and DN‐DETA hydrogels. The results presented herein have also confirmed that the immunoregulatory role of SPD can translate into faster and more natural healing of both acute wounds and diabetic wounds. Hence, we have demonstrated that the local administration of SPD in implantable biomaterials affords a simple but elegant approach to attenuate FBR induced by implants and treat chronic refractory wounds. Our work may inspire more chemists to adopt SPD in their design of implantable biomaterials.

## 5 Experimental Section

Materials and methods are provided in Supporting Information.

## Conflict of Interest

Thomas M. Roberts is a founder of Crimson Biotech, Crimson Biotech China, and Geode Therapeutics, and is a member of the scientific advisory boards of Crimson Biotech, and Geode Therapeutics.

## Author Contributions

Q.W., R.Y., W.F., L.W., S.L., B.W., and Z.L. conceived and designed experiments. Q.W., R.Y., W.F., L.W., J.Z., T.C., Q.L., X.P., Y.Z., W.Z., S.Z. performed the surgeries and the in vitro and in vivo experiments. Q.W., R.Y., L.W., J.Y., S.L., and Z.L. analyzed and interpreted data. Q.W., W.F., S.L., T.M.R., and Z.L. wrote and revised the manuscript. S.L. and Z.L. provided administrative and material support. J.Y., S.L., and Z.L. supervised and coordinated all aspects of the work.

## Supporting information

Supporting Information

Supporting Information

Supporting Information

Supporting Information

Supporting Information

Supporting Information

## Data Availability

The data that support the findings of this study are available from the corresponding author upon reasonable request.
