# Supplementary material for: Spermidine‐Functionalized Injectable Hydrogel Reduces Inflammation and Enhances Healing of Acute and Diabetic Wounds In Situ
Source: Adv Sci (Weinh). 2024 Apr 11;11(22):2310162. doi: 10.1002/advs.202310162 (PMC11165486; doi:10.1002/advs.202310162)
Supplement: Supplementary file 1 — Supporting Information [file ADVS-11-2310162-s005.pdf]

ADVANCED SCIENCE
Open Access

Supporting Information

for *Adv. Sci.*, DOI 10.1002/advs.202310162

Spermidine-Functionalized Injectable Hydrogel Reduces Inflammation and Enhances Healing of Acute and Diabetic Wounds In Situ

Qianqian Wu, Runjiao Yang, Wenxuan Fan, Li Wang, Jing Zhan, Tingting Cao, Qiming Liu, Xianshu Piao, Yinghui Zhong, Wenxian Zhao, Shuhan Zhang, Jiaao Yu, Song Liang, Thomas M. Roberts, Bingdi Wang and Zhenning Liu**

Supplementary Materials for

Spermidine-functionalized injectable hydrogel reduces inflammation and enhances healing of acute and diabetic wounds *in situ*

Qianqian Wu[1], Runjiao Yang[1], Wenxuan Fan[1], Li Wang[1], Jing Zhan[2], Tingting Cao[2], Qiming Liu[1], Xianshu Piao[1], Yinghui Zhong[1], Wenxian Zhao[1], Shuhan Zhang[1], Jiaao Yu[3], Song Liang[1], Thomas M. Roberts[4,5], Bingdi Wang*[,1], Zhenning Liu*[,1]

[1]Key Laboratory of Bionic Engineering (Ministry of Education), Jilin University, Changchun, 130022, China
[2]Department of Gastroenterology, The First Hospital of Jilin University, Jilin University, Changchun, 130021, China
[3]Department of Burn Surgery, The First Hospital of Jilin University, Jilin University, Changchun, 130061, China
[4]Department of Cancer Biology, Dana-Farber Cancer Institute, Boston, Massachusetts, 02215, USA
[5]Department of Biological Chemistry and Molecular Pharmacology, Harvard Medical School, Boston, Massachusetts, 02215, USA

*Corresponding author email: bingdi_wang@jlu.edu.cn, liu_zhenning@jlu.edu.cn

This file includes:

1. Supplementary materials and methods
2. Supplementary figures (Figure S1- Figure S22)
3. Supplementary tables (Table S1-Table S4)
4. List of supplementary videos (Video S1- Video S5)
5. Supplementary references (1-7)

1. Supplementary materials and methods

1.1 Materials

O-carboxymethyl chitosan (OCMCS, deacetylation degree 90%) was purchased from Santa Cruz Biotechnology (USA). Gelatin methacryloyl (GMA) and lithium phenyl-2,4,6-trimethylbenzoylphosphinate (LAP, photoinitiator) were purchased from EFL (Suzhou, China). Spermidine (SPD), tetramethylbenzidine (TMB), lipopolysaccharide (LPS), and 4',6-diamidino-2-phenylindole (DAPI) were purchased from Sigma-Aldrich (USA). Diethylenetriamine (DETA) was purchased from Aladdin (Shanghai, China). 4-arm poly(ethylene glycol) benzaldehyde (4aPEG-BA, MW=10 kDa) was purchased from SINOPEG Biotechnology (Xiamen, China). Recombinant mouse interleukin-4 (IL-4) was purchased from PeproTech (USA). Dulbecco's modified Eagle's medium (DMEM), fetal bovine serum (FBS) and penicillin-streptomycin (P-S) were purchased from Gibco/ThermoFisher Scientific (USA). High glucose complete DMEM was purchased from SUNNCELL Biotechnology (Wuhan, China). Cell Counting Kit-8 (CCK-8) and Calcein-AM/PI (Live/Dead) Double Staining Kit were purchased from Dojindo Chemical Technology (Shanghai, China). 2,2-diphenyl-1-picrylhydrazyl (DPPH), dihydroethidium (DHE) and Reactive Oxygen Species Assay Kit (ROS Assay Kit) were purchased from Beyotime (Shanghai, China).

1.2 Fabrication of DN, DN-SPD and DN-DETA hydrogels

The hydrogels were obtained by mixing the precursor solution with 4aPEG-BA solutions at room temperature. Briefly, a 12 wt% GMA solution was prepared by dissolving GMA in phosphate-buffered saline (PBS) containing photoinitiator (LAP, 0.5% w/v) at 60 °C and a 3 wt% OCMCS solution was prepared by dissolving OCMCS in PBS at room temperature. Then GMA and OCMCS solutions were mixed at 1:1 ratio to obtain the precursor solution. The 4aPEG-BA solution was prepared by dissolving 0.2 g of the polymer in 1.0 mL PBS. The OCMCS/GMA precursor solution was mixed with 4aPEG-BA solution to form the single network hydrogel (SN) based on Schiff base reaction. Then the SN hydrogel was irradiated with blue light (405 nm, 25 mW/cm^2) for 30 s to form the second network by photo- crosslinking GMA. The double network hydrogel without SPD or DETA was named as DN hydrogel. The hydrogels

containing different concentrations of SPD or DETA (denoted as DN-SPD or DN-DETA respectively) were prepared by similar protocol except that different volumes of SPD or DETA were introduced into the OCMCS/GMA precursor solution. The SN hydrogels with SPD or DETA before photo-crosslinking were designated as SN-SPD and SN-DETA respectively.

1.3 Morphological and chemical characterizations

Scanning electron microscopy (SEM) images were obtained on a XL-30 ESEM FEG FEI COMPANYTM electron microscope (USA). The pore sizes of hydrogels were measured by ImageJ software. Fourier transform infrared (FT-IR) spectra were recorded on a Shimadzu RF-5301PC spectrometer in the transmission mode using KBr pellets of the samples.

1.4 Mechanical tests

The mechanical properties of hydrogels were measured by compression tests. Briefly, hydrogels were prepared as cylinders (10 mm in diameter and 7 mm in height) and compressed at a speed of 0.01 $mm \cdot s^{-1}$ to a maximum strain of 70% with a universal test machine (Instron 1121, Instron, USA). The compressive moduli were calculated from the slopes of the linear region (0-10%) in stress-strain curves as previously described [1].

1.5 Swelling ratio

For the analysis of swelling property, hydrogels were immersed in PBS (pH = 7.4) for 24 h (or as otherwise indicated) at 37 °C, and the weights of swollen hydrogels (W_t) were recorded after removing residual PBS with a filter paper. Subsequently, the swollen hydrogels were lyophilized and weighted to obtain W_0. Swelling ratio was calculated according to Eq. (S1):

$$Swelling\ ratio = (W_t - W_0) / W_0 \qquad (S1)$$

where W_t and W_0 represent the weight of swollen hydrogels at each timepoint and the weight of lyophilized hydrogels, respectively.

1.6 Self-healing and injectability assay

The rheological properties of SN-SPD hydrogel were characterized on an Anton Paar MCR302 rheometer in an oscillatory mode. Samples were placed in a plate with a

diameter of 25 mm and strain sweep tests were carried out at a fixed frequency of 1 rad·s^{-1} within a strain range of 1% to 500%. Subsequently, continuous oscillatory step-strain experiments were conducted at a constant frequency (1 rad·s^{-1}) with alternating cycles between 1% and 500% strain to examine the self-healing property of the hydrogel. To further illustrate the self-healing ability of the hydrogel, two star-shaped hydrogels were made in two colors: one is blue and the other is yellow. The two stars were cut into halves and swapped. The halves of different colors were placed next to each other without any external force and incubated at 37 °C. To demonstrate the injectability, the <u>SN-SPD</u> hydrogel was injected through a syringe equipped with a 22G needle to print the letters of "MMB".

1.7 Tissue adhesion assays

The tissue adhesion of <u>DN-SPD</u> hydrogel was studied on a pig skin purchased from a local market. First, for qualitative assay, <u>SN-SPD</u> hydrogel was injected onto the pig skin surface and then irradiated with blue light (405 nm, 25 mW/cm^2) for 30 s to form <u>DN-SPD</u> hydrogel *in situ*. The adhesion ability of the <u>DN-SPD</u> hydrogel was evaluated by macroscopic observation of detachment under torsion. Second, for semi-quantitative assay, <u>SN-SPD</u> hydrogel was injected onto one end of the pig skin, covered with a glass slide, and then irradiated with blue light (405 nm, 25 mW/cm^2) for 30 s through the glass slide to form <u>DN-SPD</u> hydrogel between the pig skin and the glass slide. Different weights were loaded onto the other end of the pig skin until significant detachment occurred, in order to evaluate the tissue adhesion strength.

1.8 Chemical antioxidant assays

First, the antioxidant capacity of hydrogels was evaluated by DPPH radical scavenging assay [2]. Briefly, DPPH solution was prepared at a concentration of 100 μM in anhydrous ethanol. Then, 2 mL DPPH solution was incubated with 150 μL hydrogels in dark at 37 °C. DPPH ethanol solution alone was used as the control. After incubation of different time, the absorbance of DPPH solution was measured by a UV-Vis spectrometer at 517 nm. The scavenging efficiency of DPPH radical was calculated by the following Eq. (S2):

$$DPPH\ scavenging\ (\%) = (A_b - A_s) / A_b \times 100\% \quad (S2)$$

where A_b and A_s represent the absorbances of the DPPH alone and the DPPH solution after incubation with hydrogels, respectively.

Second, the ·OH scavenging ability was investigated for free SPD, free DETA and different hydrogels. ·OH was generated through Fenton reactions according to a previous report with minor modifications [3]. Briefly, for free SPD and DETA, 100 μL SPD or DETA solutions at different concentrations were mixed with $FeSO_4$ solution (1 mM, 500 μL) and H_2O_2 solution (100 mM, 500 μL), and then incubated at 37 °C for 1 h. For hydrogels, 150 μL hydrogels were incubated with the mixture of $FeSO_4$ solution (1 mM, 500 μL) and H_2O_2 solution (100 mM, 500 μL) at 37 °C for 1 h. 100 μL supernatant was collected after cooling to room temperature and mixed with TMB (10 mM, 100 μL). After 10 min, the concentration of ·OH was measured by the absorbance at the wavelength of 650 nm using a microplate reader. The scavenging efficiency of ·OH was calculated according to Eq. (S3):

$$\cdot OH\ scavenging\ (\%) = (A_b - A_s) / A_b \times 100\% \quad (S3)$$

where A_b and A_s represent the absorbances of the blank control and the solution incubated with free SPD, free DETA or different hydrogels, respectively.

Third, the H_2O_2 scavenging efficiency of hydrogels was characterized as previously described [4]. Briefly, 150 μL hydrogels were incubated with 500 μL H_2O_2 solution (1 mM) at 37 °C. PBS was added to 500 μL H_2O_2 solution (1 mM) as the control. After incubation of different time, 20 μL supernatant was collected and mixed with 100 μL $Ti(SO_4)_2$ solution (5 mM in 3 M H_2SO_4). After 30 min, the concentration of H_2O_2 was determined by the absorbance at the wavelength of 405 nm using a microplate reader. The scavenging efficiency of H_2O_2 was calculated according to Eq. (S4):

$$H_2O_2\ scavenging\ (\%) = (A_b - A_s) / A_b \times 100\% \quad (S4)$$

where A_b and A_s represent the absorbances of the PBS control and the solution incubated with different hydrogels, respectively.

1.9 Degradation assay *in vitro*

The initial weights (W_i) of freeze-dried hydrogels were measured. Then the freeze-dried hydrogels were immersed in PBS and incubated at 37 ºC. At different timepoints, the remaining weights of hydrogels (W_t) were recorded. The remaining weight percentages of hydrogels were calculated according to Eq. (S5):

$$\textit{Weight remaining}\ (\%) = (W_t / W_i) \times 100\% \qquad \text{(S5)}$$

where W_t and W_i represent the weight of the remaining hydrogel at each timepoint and the initial weight of the hydrogel, respectively.

1.10 Cell culture

Mouse fibroblasts (L929) and macrophages (RAW 264.7) were purchased from Procell Life Science & Technology (Wuhan, China). L929 cells were cultured in DMEM supplemented with 10% FBS and 1% P-S at 37 ℃ with 5% CO_2. RAW 264.7 cells were cultured in high glucose complete DMEM (SUNNCELL, Wuhan, China) at 37 ℃ with 5% CO_2.

1.11 Cell viability and proliferation assays

For cell viability assay, L929 and RAW 264.7 cells were seeded in the lower chambers of 24-well transwell plates with 0.4 μm pore size (Corning, USA) at a density of 5×10^4 cells/well and incubated overnight. Subsequently, 150 μL hydrogels were added to the upper chambers of transwell plates. The well without hydrogel was used as the blank control. After incubation at 37 °C for 24 h, the upper chambers were removed. Cells were counted with CCK-8 kits by following manufacturer’s protocol and the absorbance at the wavelength of 450 nm was recorded on a microplate reader (MOLECULAR DEVICES, SpectraMax Absorbance Reader, CMax Plus, BK-200 L96C).

The proliferation of L929 cells with hydrogels was examined by two methods. For the method of coculturing in transwell plates, L929 cells were seeded in the lower chambers of 24-well transwell plates with 0.4 μm pore size (Corning, USA) at a density of 5×10^4 cells/well. 150 μL hydrogels were added to the upper chambers of transwell plates. The well without the upper chamber was used as the blank control. After incubation at 37 °C for 24 h and 72 h, the upper chambers were removed. Cells were

washed with PBS twice and then incubated with Calcein-AM/PI solution at 37 ℃ for 15 min. Cells were observed by a fluorescence microscope (Olympus IX53, Japan).

For the method of coculturing on the surface of hydrogels, the hydrogels (height ~ 1.5 mm) were prepared in 24-well plates under sterile condition and L929 cells were seeded on the surface of hydrogels at a density of 5×10^4 cells/well. After incubation of indicated periods, CCK-8 assay was carried out by following manufacturer's protocol and the absorbance at the wavelength of 450 nm was recorded.

1.12 Hemolysis assay

The human blood used in the hemolysis assay was collected from the corresponding author, Dr. Zhenning Liu. Hemocompatibility of hydrogels was evaluated by hemolysis assay as previously described [5]. Briefly, red blood cells (RBCs) were obtained by centrifuging fresh human blood at 2000 rpm for 10 min and washed with 0.9% NaCl solution five times. Purified RBCs were resuspended in 0.9% NaCl solution at a rough concentration of 5% (v/v). Then, 200 µL RBC suspension was mixed with 100 µL homogenized hydrogel liquid and 700 µL 0.9% NaCl solution in 1.5 mL tube. After incubation at 37 °C for 1 h, the mixtures were centrifuged at 2000 rpm for 10 min and the absorbance of the supernatant was measured at the wavelength of 540 nm using a microplate reader. Triton X-100 (0.1%) and 0.9% NaCl solution served as the positive and negative controls, respectively. The tests were done in triplicates for each sample. The hemolytic ratio (HR) was calculated according to Eq. (S6):

$$HR\ (\%) = [(OD_s - OD_n) / (OD_p - OD_n)] \times 100\% \qquad \text{(S6)}$$

where OD_s, OD_p, and OD_n represent the absorbances of sample, positive control and negative control, respectively.

1.13 Scratch healing assay *in vitro*

The scratch healing assay was performed in 6-well transwell plates. L929 cells were seeded in the lower chambers and allowed to form a confluent monolayer. Cells were scratched with a sterilized 200 µL pipette tip to form one linear wound and washed with PBS to remove cell debris. Then fresh culture medium without serum was added, and cells were incubated with or without hydrogels in the upper chamber. The wound sizes at 0, 12, 24 and 48 h were photographed by a microscope (IX53, Olympus, Japan).

1.14 Biological antioxidant assays

Dihydroethidium (DHE) and 2',7'-dichlorofluorescin diacetate (DCFH-DA) were used to evaluate the levels of intracellular superoxide anion and reactive oxygen species (ROS), respectively [6]. Briefly, L929 cells were seeded in the lower chambers of 24-well transwell plates at a density of 5 × 10^4 cells/well and incubated at 37 °C for (overnight). The confluent L929 cells were pretreated with hydrogels in upper chambers for 12 h, while the blank control was cultured without the upper chamber. Cells were treated with H_2O_2 (100 μM) for 6 h. Then the cells were washed with PBS three times, followed by incubation with DHE (5 μM) or DCFH-DA (10 μM) for 30 min. Subsequently, cells were washed with PBS three times and counterstained with DAPI for 10 min in the dark to label nuclei. The images were collected by a fluorescence microscope (Eclipse C1, Nikon, Japan) and the mean fluorescence intensity (M.F.I.) was quantified with ImageJ software.

1.15 Macrophage polarization *in vitro*

Raw 264.7 cells were seeded at a density of 5 × 10^4 cells/well in the lower chambers of 24-well transwell plates and cultured overnight. Cells were stimulated with 1 μg/mL LPS and treated with different hydrogels in the upper chambers for 48 h. Cells treated with 1 μg/mL LPS and 40 ng/mL IL-4 were used as the negative control and positive control, respectively. The blank control was without any treatment.

1.16 Immunofluorescence (IF)

RAW 264.7 cells were cocultured with different hydrogels as described above and fixed on slides with 4% paraformaldehyde (PFA). Then, the cells were blocked with 3% bovine serum albumin (BSA) for 30 min at room temperature, washed with PBS for three times, and then incubated with rabbit anti-CD86 antibody (1:200, 13395-1-AP, Proteintech, USA) overnight at 4 °C. After washing with PBS, cells were incubated with Alexa Fluor 488-conjugated goat anti-rabbit IgG antibody (1:200, ab150077, Abcam, UK) for 50 minutes at room temperature. The cells were washed with PBS three times and incubated with DAPI for 10 min at room temperature to label nuclei. IF images were collected by a fluorescence microscope (Eclipse C1, Nikon, Japan) and analyzed by ImageJ software. For CD206 marker, cells were stained with rabbit anti-

CD206 antibody (1:1000, ab64693, Abcam, UK) and Cy3-conjugated goat anti-rabbit IgG antibody (1:400, GB21301, Servicebio, China) using the same protocol.

1.17 Cytokine and chemokine assays

For cells, the supernatants of RAW 264.7 culture with different treatments were collected by centrifugation at 12000 rpm for 10 min at 4 ℃ for enzyme-linked immunosorbent assay (ELISA) on pro-inflammatory cytokines, interleukin-6 (IL-6) and tumor necrosis factor-α (TNF-α), and anti-inflammatory cytokine, interleukin-10 (IL-10) with respective cytokine kits (Peprotech, USA).

For mouse tissues, wound tissues were weighed and added to homogenization buffer, followed by mechanical homogenization in ice-water bath to prepare suspensions at a rough concentration of 10% (w/v). The suspensions were centrifuged at 2500 rpm for 10 min at 4 °C to collect the supernatants. The concentrations of TNF-α, IL-10, IL-6, interleukin 1β (IL-1β), and transforming growth factor-β (TGF-β) in the supernatants were measured with respective ELISA kits (Invitrogen, USA).

ELISAs were performed according to the manufacturers' protocols.

1.18 Real-time quantitative polymerase chain reaction (RT-qPCR)

RAW 264.7 cells were washed with precooled PBS, and then lysed with 1 mL RNA extraction reagent by pipetting up and down. Rat tissues (100 mg) were ground in 1 mL RNA extraction reagent. Total RNA was extracted by Trizol reagent (10296028, Invitrogen, USA) and then quantified by a Microvolume Spectrophotometer (NanoDrop2000, ThermoFisher, USA). Next, cDNA was synthesized by SuperScript III Reverse Transcription Kit (11752050, Invitrogen, USA). RT-qPCR was performed on a StepOnePlus Real-Time PCR System (Applied Biosystems, USA) using SYBR Select Master Mix (4472920, Invitrogen, USA). mRNA levels were normalized to the mRNA level of actin for RAW 264.7 cells and glyceraldehyde-3-phosphate dehydrogenase (GAPDH) for rat tissues. The results were analyzed by the ΔΔCt method. The sequences of RT-qPCR primers are listed in Table S1 and S2.

1.19 Immunoblotting

RAW 264.7 cells were washed with PBS for three times and then lysed with RIPA lysis buffer (G2002, Servicebio, China). The cells were collected with a cell scraper

and transferred into 1.5 mL centrifuge tubes. Cells were lysed on ice for 30 minutes and centrifuged at 12000 rpm for 10 min at 4 ℃. The supernatants were collected and the total protein concentrations were quantified with BCA protein assay kit (MD913053, Medical Discovery Leader, China). The lysates were electrophoresed in 10% SDS-PAGE (Bio-Rad, USA) and then transferred onto polyvinylidene fluoride (PVDF) membranes (Millipore, USA). After blocking with 5% nonfat milk in TBST buffer for 1 h, the PVDF membranes were incubated with primary antibodies (rabbit anti-iNOS antibody ab178945, Abcam, UK; rabbit anti-NF-κB antibody, ab32536, Abcam, UK; rabbit anti-Arg1 antibody, ab233548, Abcam, UK; rabbit anti-CD86 antibody, bs-1035R, Bioss, China; rabbit anti-CD206 antibody, bs-23178R, Bioss, China; or rabbit anti-β-actin antibody, Medical Discovery Leader, China) overnight at 4 °C. After washing with TBST buffer, the PVDF membranes were incubated with HRP-conjugated goat anti-rabbit IgG antibody (MD6553, Medical Discovery Leader, China) for 60 min at room temperature. After washing with TBST buffer for three times, the PVDF membranes were visualized with a Chemiluminescence Imaging System (ChemiScope 6000, CLINX, China).

1.20 Subcutaneous implantation in rats

All animals were housed and treated in accordance with protocols approved by the Jilin GENET-MED Biotechnology (Approval No: IACUC-2022-001. The original approval document in Chinese is available from the authors). Before surgery, male SD rats (200-220 g, 8 weeks, n = 16) were anesthetized by intraperitoneal injection of 2% tribromoethanol (10 mL/kg). Then the dorsa of rats were shaved and sterilized with 75% ethanol. Four linear incisional skin wounds about 10 mm were generated using surgical scissors, and then cylindrical DN, DN-SPD and DN-DETA hydrogels (diameter ≈ 10 mm, height ≈ 3 mm) were embedded inside three wounds on the same rat, whereas the fourth wound without any hydrogel was used as the sham control. Each kind of hydrogels was implanted into twelve animals. After implantation, the incisions were closed by surgical sutures. Three rats were sacrificed on postsurgical Day 3, 7, 14 and 21 (n = 12 = 3 × 4). The remaining hydrogels together with surrounding tissues were photographed and collected for future experiments. In addition, another 3 rats were

implanted with 4 hydrogels (diameter ≈ 10mm, height ≈ 3mm) of the same kind to examine the toxicity on various organs. These 3 rats represent DN, DN-SPD, and DN-DETA hydrogels, whereas the last rat that was cut to make four incisions but sutured without implanted hydrogels served as the sham control. These 4 rats were sacrificed on postsurgical Day 21, and their heart, liver, spleen, lung and kidney were harvested and fixed in 4% PFA for hematoxylin and eosin (H&E) staining.

1.21 Acute wound healing in normal mice

The acute wound healing experiments were carried out on male C57BL/6 mice (20-25 g, 6-8 weeks) with full-thickness skin defects. Briefly, all mice (n = 27) were anesthetized by intraperitoneal injection of 2% tribromoethanol (0.02 mL/g). Then the dorsa were shaved and sterilized with 75% ethanol. Two full-thickness cutaneous wounds were generated with a circular skin biopsy puncher (8 mm in diameter). Subsequently, the wounds were treated with commercial product (Duo DERM), DN, DN-SPD 100, DN-SPD 250, or DN-DETA 250 hydrogels, whereas the wound treated with PBS was used as the blank control. Each treatment (including PBS) was done on 9 wounds. Then the wounds were covered with 3M Tegaderm Transparent Film (1624W, USA), which were removed after two days. On postsurgical Day 0, 3, 7 and 12, the wounds were photographed and the wound areas were measured by ImageJ software. Wound closure rates were calculated according to Eq. (S7):

$$\textit{Wound closure}\ (\%) = (A_0 - A_t) / A_0 \times 100\% \qquad \text{(S7)}$$

where A_0 and A_t represent the wound areas on Day 0 and Day t, respectively.

Nine mice were sacrificed on postsurgical Day 3, 7, and 12 (n = 27 = 9 × 3), which contained 18 wounds for 6 treatments in triplicates.

1.22 Diabetic wound healing *in situ*

The *in situ* diabetic wound healing experiments were carried out on male *db/db* mice (35-40 g, 6-8 weeks) with full-thickness skin defects. Briefly, all mice (n = 24) were anesthetized by intraperitoneal injection of 2% tribromoethanol (0.02 mL/g). Then the dorsa were shaved and sterilized with 75% ethanol. Two full-thickness cutaneous wounds were generated with a circular skin biopsy puncher (8 mm in diameter). Subsequently, SN, SN-SPD or SN-DETA hydrogels were injected onto the wounds and

irradiated with blue light (405 nm, 25 mW/cm^2) to form *in situ* DN, DN-SPD or DN-DETA hydrogels, respectively. The wound treated with PBS and irradiated with blue light was used as the blank control. Each treatment (including PBS) was done on 12 wounds. On postsurgical Day 0, 3, 7, 10, 14, 18 and 21, the wounds were photographed and the wound areas were measured by ImageJ software. Wound closure rates were also calculated according to Eq. (S7). Six mice were sacrificed on postsurgical Day 3, 7, 14, and 21 (n = 24 = 6 × 4), which contained 12 wounds for 4 treatments in triplicates.

For the animal experiments in this work, including the subcutaneous implantation in rats, acute wound healing in normal mice and diabetic wound healing in *db*/*db* mice, the tissues collected around the implanted hydrogels or the wounds were divided for various assays. One portion of a harvested tissue was fixed in 4% PFA for 24 h, dehydrated and embedded in paraffin. The paraffin-embedded samples were sliced into 5-μm thick sections for histological analyses, including H&E staining, Masson's trichrome staining, immunohistochemistry (IHC) and immunofluorescence (IF). The remaining portion of the tissue was immediately frozen in liquid nitrogen after collection, and then stored at -80 °C for ELISA and RT-qPCR experiments.

H&E and Masson's trichrome stainings were performed in accordance with standard or published protocols [7]. Images were collected with a microscope (Eclipse C1, Nikon, Japan).

1.23 Immunohistochemistry (IHC) and immunofluorescence (IF)

Antigen recovery and endogenous peroxidase elimination were performed on paraffin-embedded tissues before immunostaining. For IHC, samples were blocked with 3% BSA for 30 min, and then incubated with primary antibodies overnight at 4 °C. After washing with PBS three times, samples were incubated with HRP-conjugated goat anti-rabbit IgG antibody (1:400, 5220-0336, Seracare, USA) at room temperature for 50 min. After washing with PBS three times, freshly prepared diamindbenzidine (DAB) solution was added for color development. Finally, samples were incubated with hematoxylin for 3 min to counterstain nuclei. IHC images were collected by a microscope (E100, Nikon, Japan) and analyzed by ImageJ software. The primary antibodies used in IHC were listed in Table S3.

For single IF staining, samples were blocked with 3% BSA for 30 min and incubated with primary antibodies overnight at 4 °C. After washing with PBS three times, samples were incubated with secondary antibodies for 50 min at room temperature. After washing with PBS three times, samples were incubated with DAPI for 10 min in the dark to label nuclei.

For double IF staining, samples were blocked with 3% BSA for 30 min and incubated with the first primary antibody overnight at 4 °C. After washing with PBST three times, samples were incubated with HRP-conjugated goat anti-rabbit IgG antibody (1:400, 5220-0336, Seracare, USA) for 50 min at room temperature. Subsequently, samples were incubated with Cy3-tyramine (1:500, G1223, Servicebio, Wuhan, China) for 20 min at room temperature and washed three times with PBST under microwave. The samples were re-blocked with 3% BSA for 10 min, and then incubated with another primary antibody overnight at 4 ℃. After washing with PBST three times, samples were incubated with HRP-conjugated goat anti-rabbit IgG antibody (1:400, 5220-0336, Seracare, USA) for 50 min at room temperature, followed by washing with PBST for three times and incubation with iF488-tyramine (1:500, G1231, Servicebio, Wuhan, China) for 20 min in the dark. After washing with PBS three times, samples were incubated with DAPI for 10 min in the dark to label nuclei.

IF staining images were collected by a fluorescence microscope (Eclipse C1, Nikon, Japan) and analyzed by ImageJ software. The antibodies used in IF were listed in Table S4.

1.24 Statistical analysis

Data are presented as mean ± standard deviation (SD) and analyzed by Origin 2018 or GraphPad Prism 8.0 software. Statistical significance was determined by using one-way ANOVA with Tukey's multiple comparisons (GraphPad Prism 8.0), and is denoted as *: $P < 0.05$, **: $P < 0.01$, and ***: $P < 0.001$, N.S.: not significant.

2. Supplementary figures

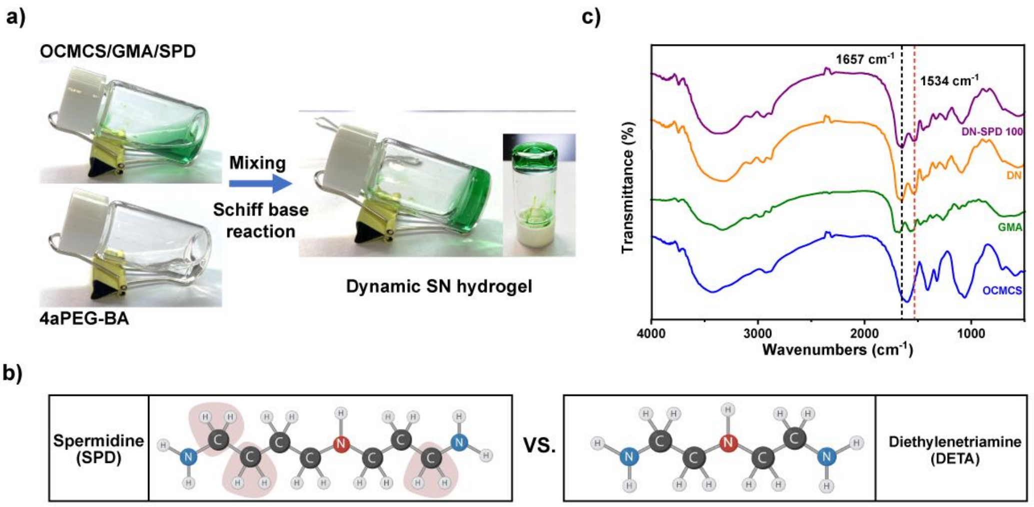

Figure S1. a) Photographs of the preparation of SN hydrogel with dynamic single network. **b)** Chemical structures of SPD and DETA. **c)** FT-IR spectra of OCMCS, GMA, and two hydrogels (DN and DN-SPD 100).

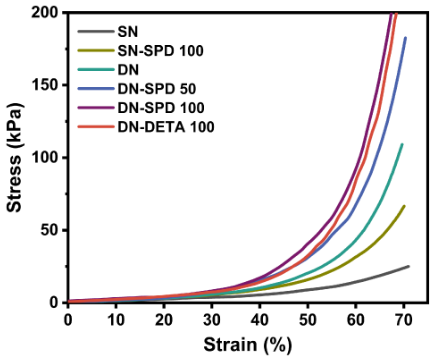

Figure S2. Stress-strain curves of compression tests for hydrogels.

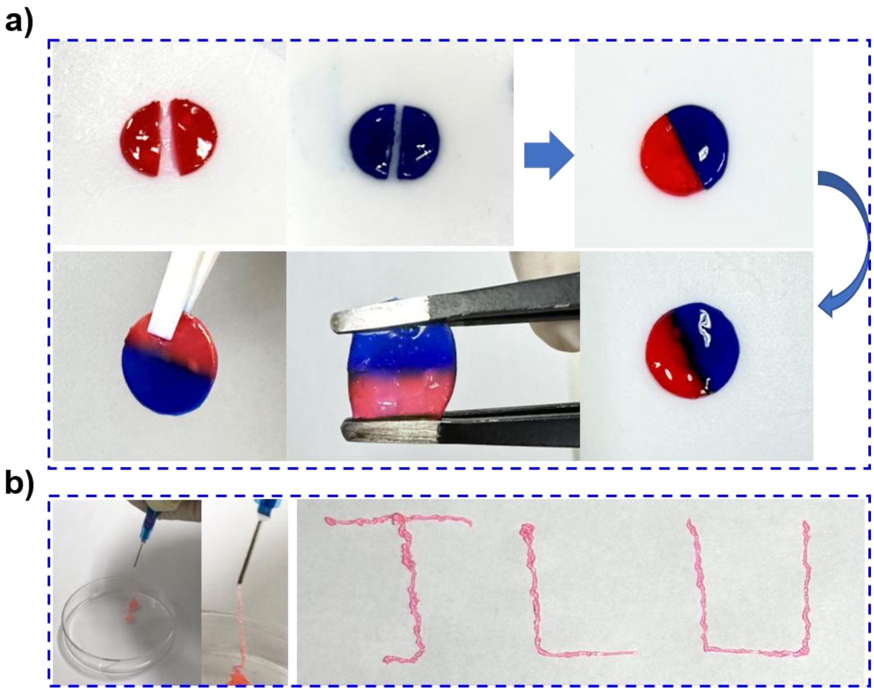

Figure S3. Photographs showing self-healing **(a)** and injectability **(b)** of SN-SPD 250 hydrogels.

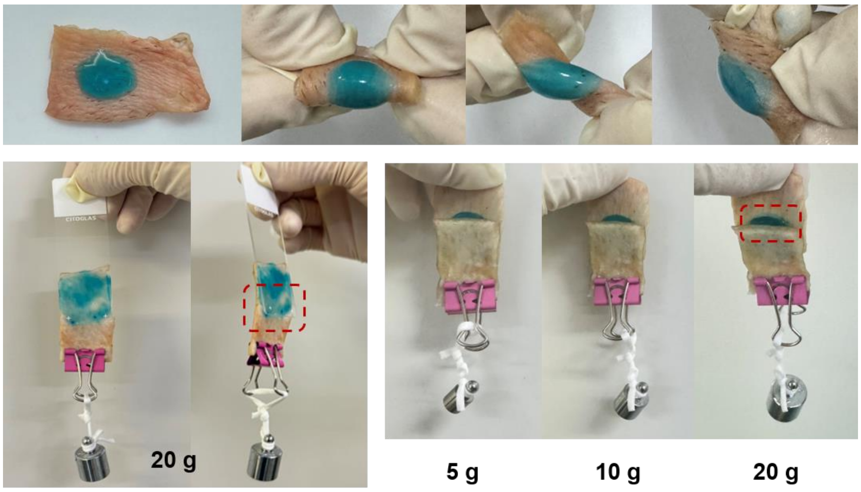

Figure S4. Photographs of SN-SPD 100 hydrogels formed on pig skin subjected to various forces.

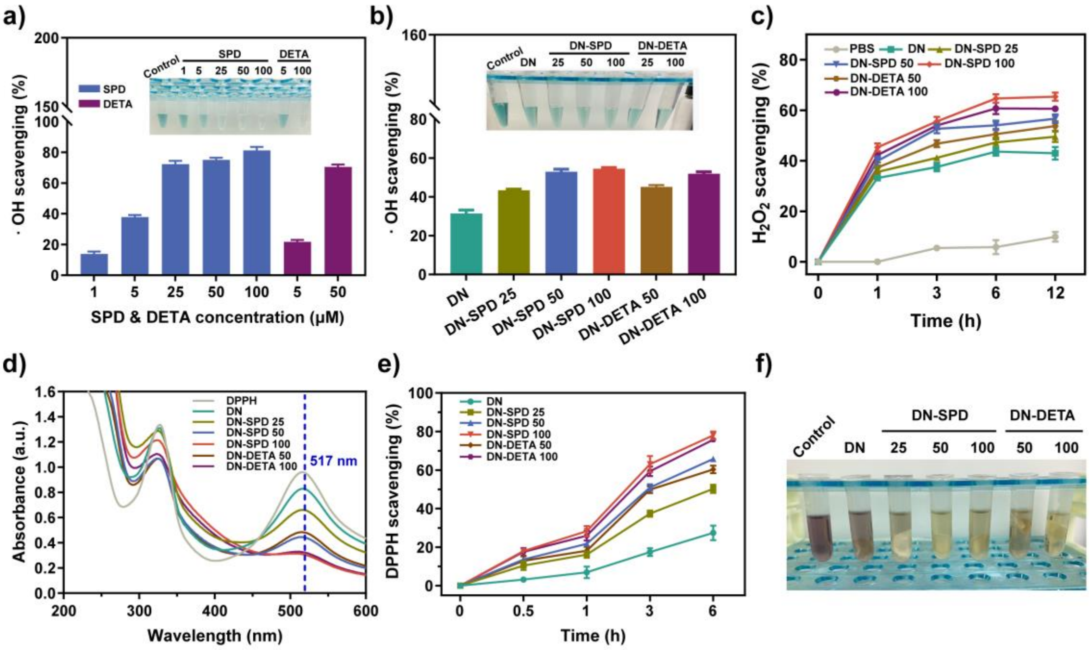

Figure S5. Antioxidant properties of hydrogels in chemical experiments. a) ·OH scavenging efficiencies for free SPD and DETA. **b)** ·OH scavenging efficiencies for various hydrogels. **c)** H_2O_2 scavenging efficiencies for various hydrogels treated with H_2O_2 for different times. **d)** UV-Vis spectra of DPPH after 6 h incubation with various hydrogels. **e)** DPPH scavenging efficiencies for various hydrogels at different timepoints. **f)** Photograph of DPPH solution after 6 h incubation with various hydrogels. Data are presented as mean ± SD (n = 3) in **(a-c)** and **(e)**.

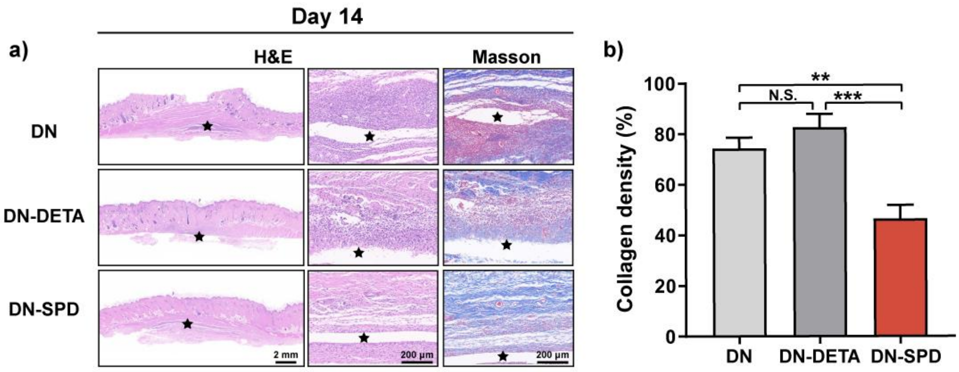

Figure S6. a) H&E and Masson's stainings of hydrogels on Day 14. **b)** Quantified collagen density within 100 microns from the implanted hydrogels. Data are presented as mean ± SD (n = 3) in **(b)**. Statistical significance was determined by using one-way

ANOVA with Tukey's multiple comparisons (GraphPad Prism 8.0). *: $P < 0.05$; **: $P < 0.01$; ***: $P < 0.001$; N.S.: not significant.

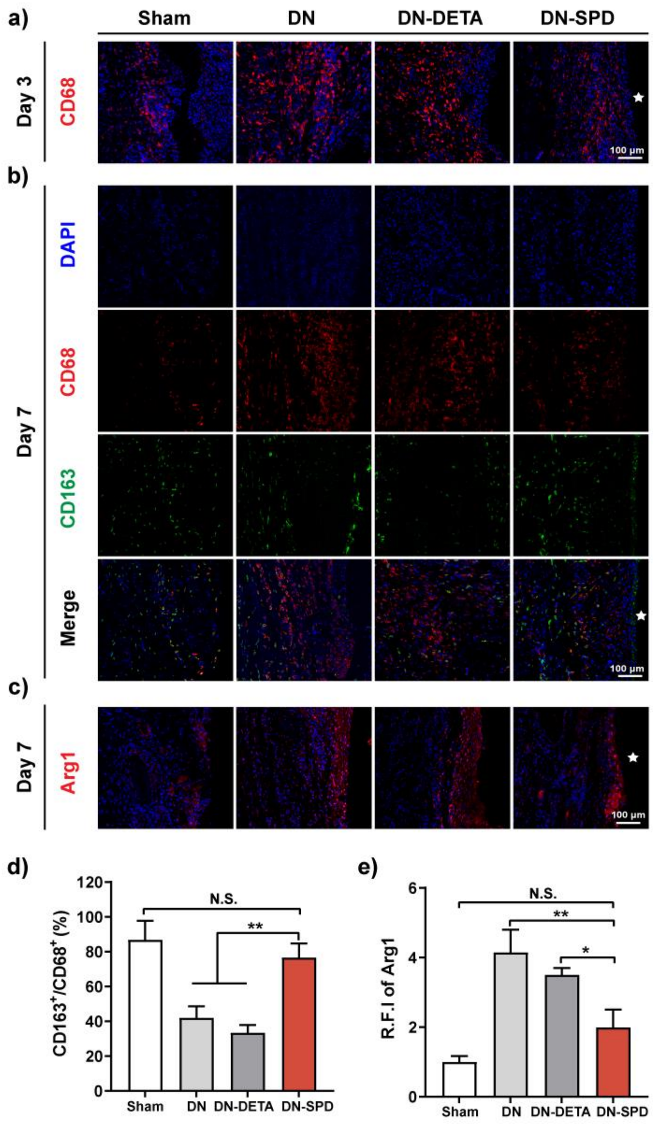

Figure S7. a) Immunofluorescent staining of CD68 on postsurgical Day 3. **b, c)** Double immunofluorescent staining of CD68/CD163 **(b)** and immunofluorescent staining of Arg1 **(c)** on postsurgical Day 7. **d)** Quantified ratios of $CD163^+/CD68^+$. **e)** Quantified relative fluorescence intensities (R.F.I.) of Arg1 staining. Data are presented as mean ± SD (n = 4) in **(d)** and **(e)**. Statistical significance was determined by using one-way ANOVA with Tukey's multiple comparisons (GraphPad Prism 8.0). *: $P < 0.05$; **: $P < 0.01$; ***: $P < 0.001$; N.S.: not significant.

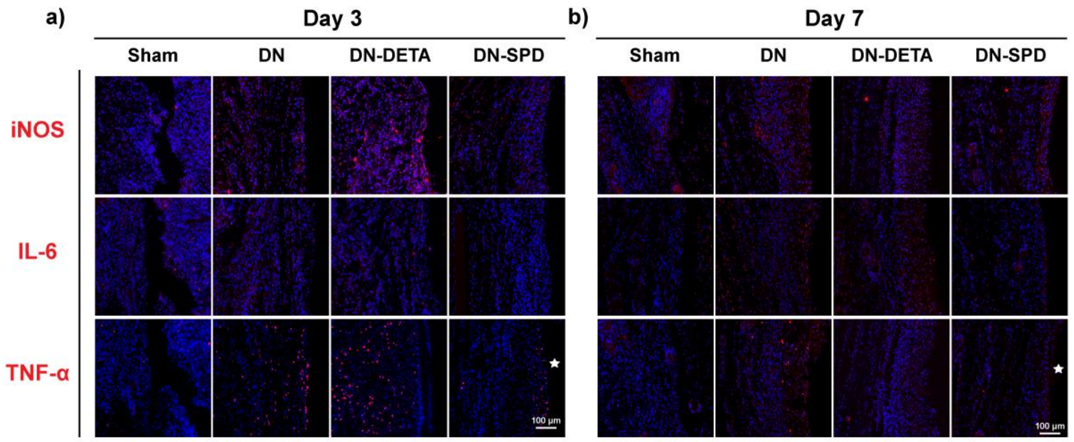

FigureS8. Immunofluorescent stainings of iNOS, IL-6 and TNF-α in tissues surrounding hydrogels on postsurgical Day 3 **(a)** and Day7 **(b)**.

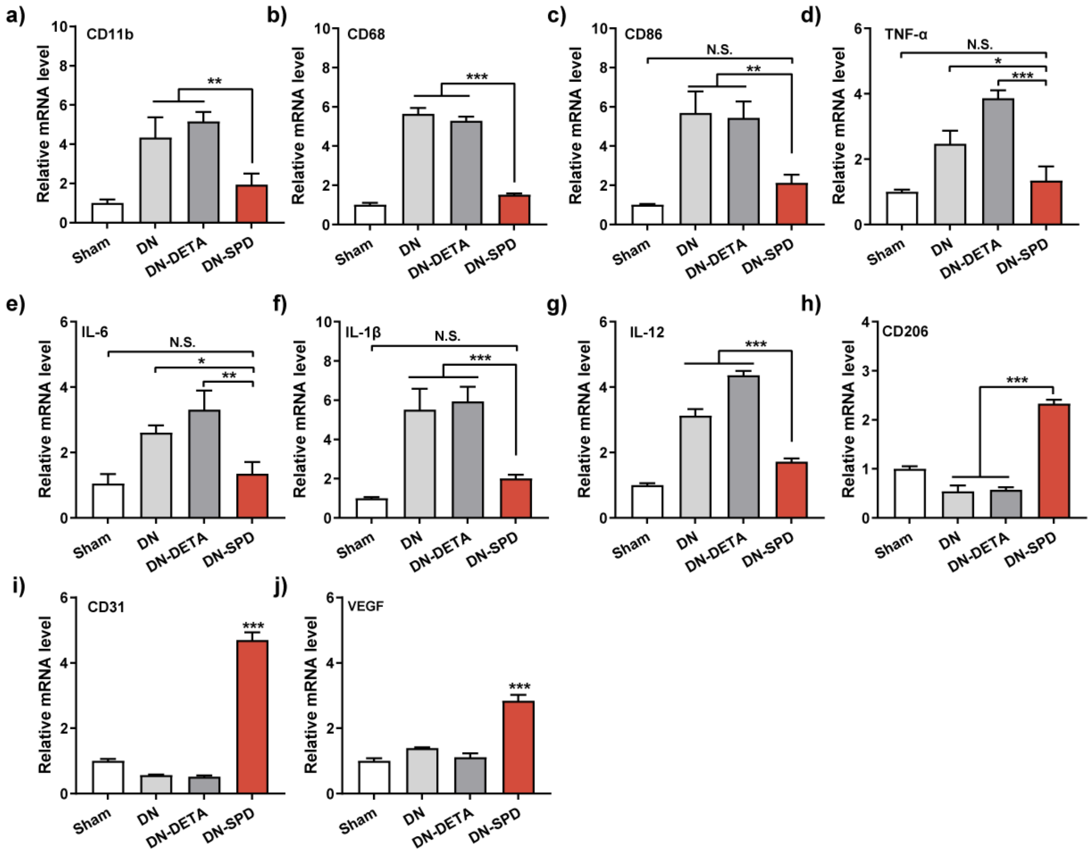

Figure S9. a-j) Relative mRNA levels of CD11b **(a)**, CD68 **(b)**, CD86 **(c)**, TNF-α **(d)**, IL-6 **(e)**, IL-1β **(f)**, IL-12 **(g)**, CD206 **(h)**, CD31 **(i)**, and VEGF **(j)** in tissues surrounding different hydrogels. Data are presented as mean ± SD (n = 3). Statistical significance was determined by using one-way ANOVA with Tukey's multiple comparisons (GraphPad Prism 8.0). *: $P < 0.05$; **: $P < 0.01$; ***: $P < 0.001$; N.S.: not significant.

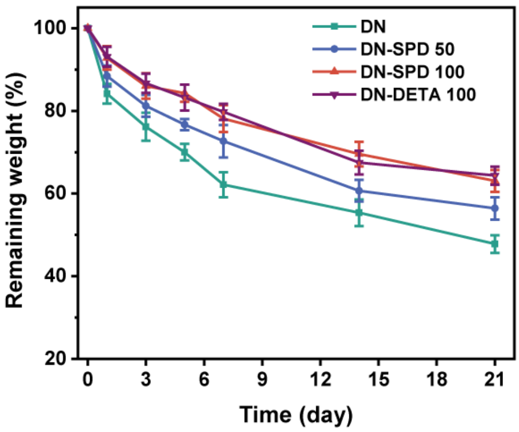

Figure S10. Remaining weights of various hydrogels after immersion in PBS (pH=7.2-7.4) for different times at 37 °C. Data are presented as mean ± SD (n = 3).

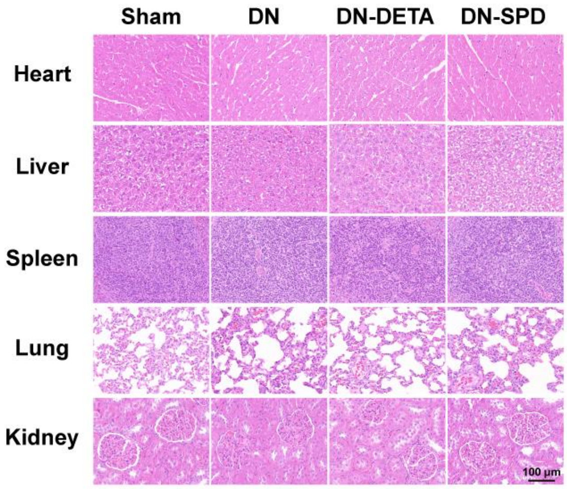

Figure S11. H&E staining of major organs (heart, liver, spleen, lung and kidney) from SD rats implanted with different hydrogels for 21 days.

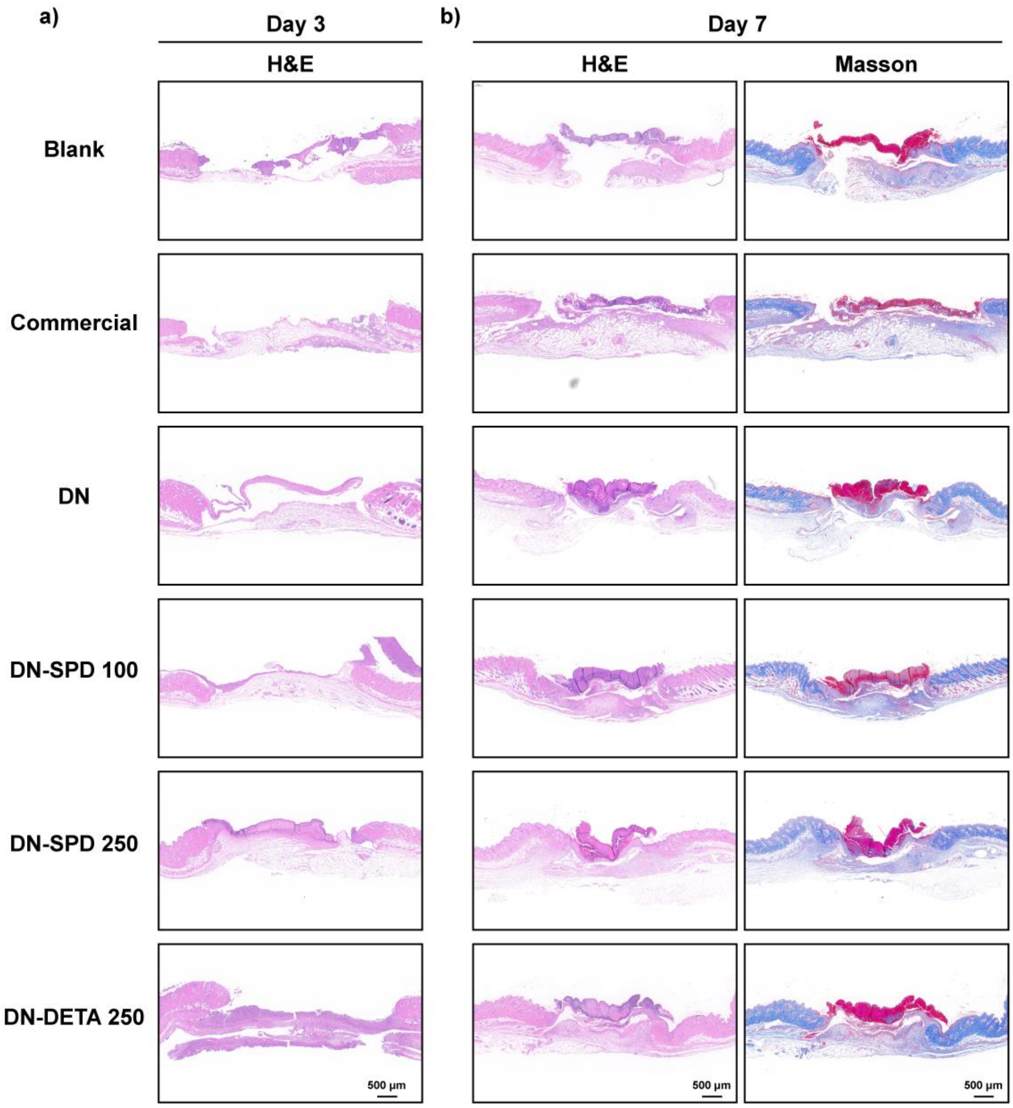

Figure S12. a) H&E staining of wounds on Day 3. **b)** H&E and Masson's trichrome stainings of wounds on Day 7.

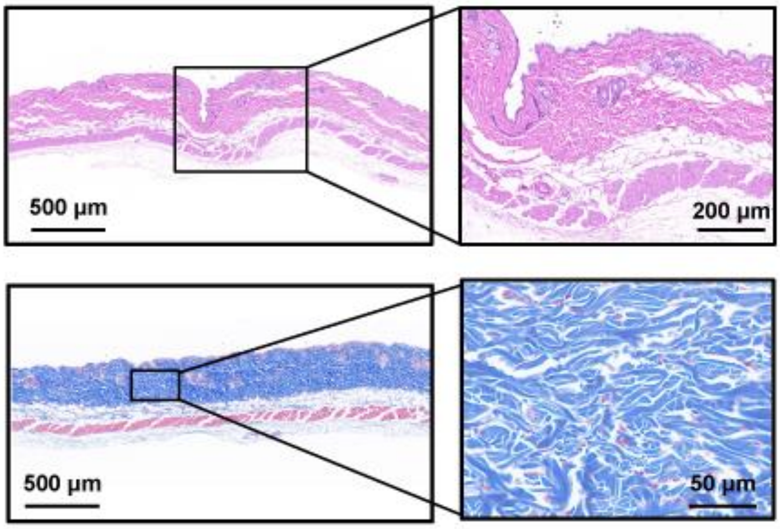

Figure S13. H&E and Masson's trichrome stainings of native mouse skin.

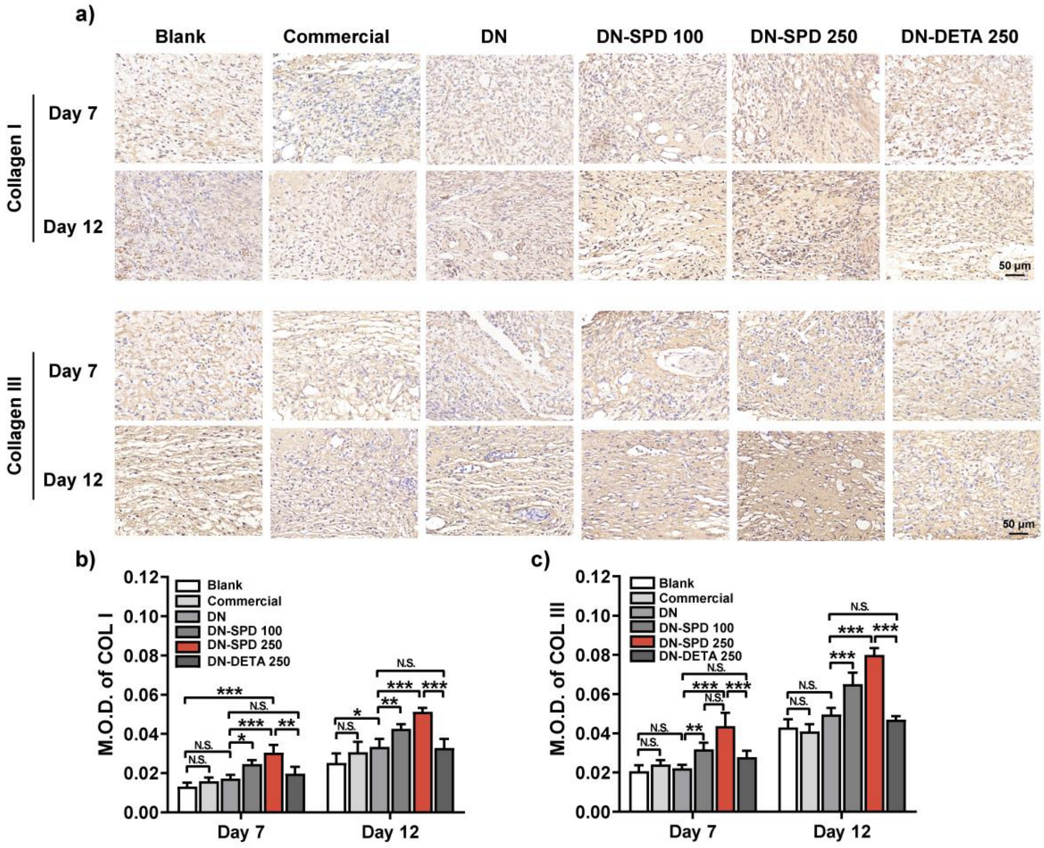

Figure S14. a) Immunohistochemical stainings of collagen I and collagen III in wound tissues on Day 7 and 12. **b, c)** Quantified mean optical densities (M.O.D.) of collagen I **(b)** and collagen III **(c)**. Data are presented as mean ± SD (n = 4) in **(b)** and (**c**). Statistical significance was determined by using one-way ANOVA with Tukey's

multiple comparisons (GraphPad Prism 8.0). *: $P < 0.05$; **: $P < 0.01$; ***: $P < 0.001$; N.S.: not significant.

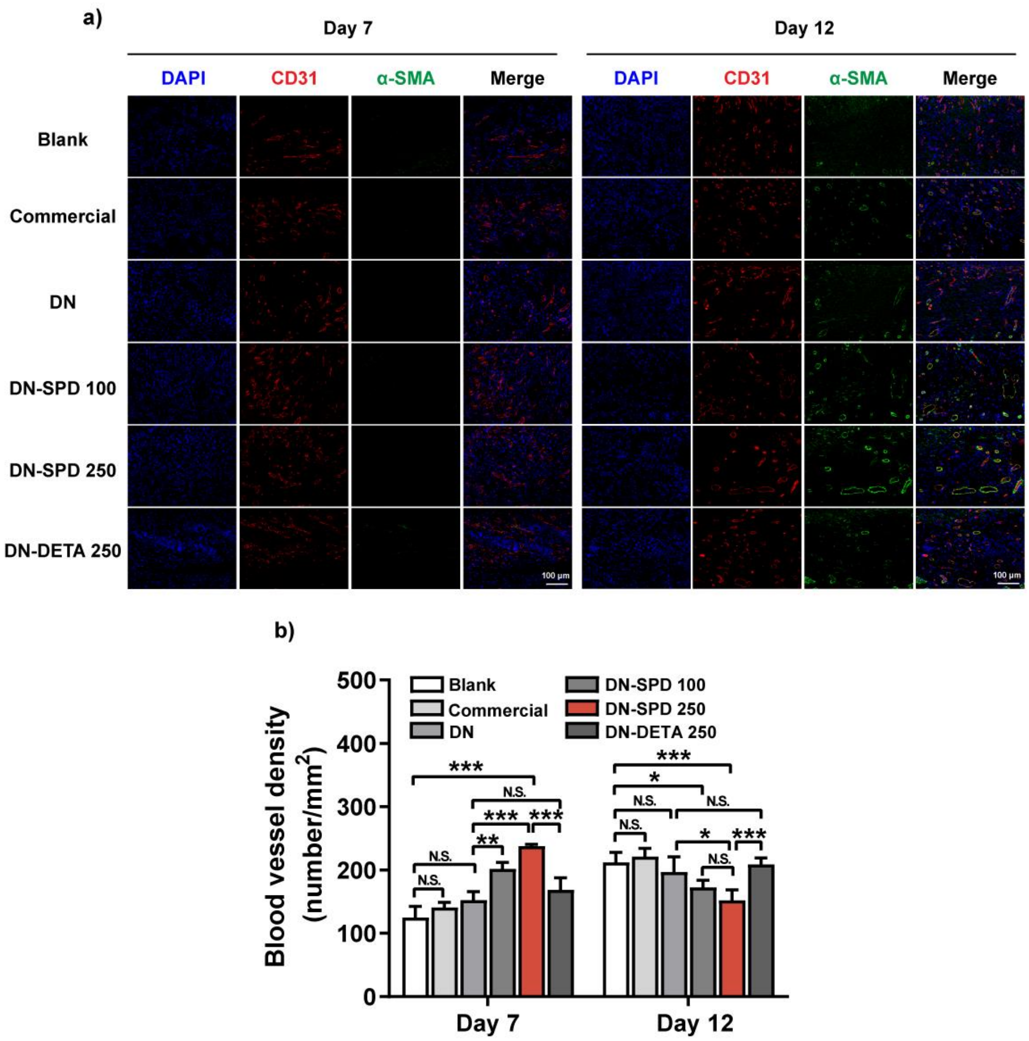

Figure S15. a) Double immunofluorescent staining of CD31/α-SMA on Day 7 and 12. **b)** Blood vessel density quantified of by CD31 staining. Data are presented as mean ± SD (n = 3) in **(b)**. Statistical significance was determined by using one-way ANOVA with Tukey's multiple comparisons (GraphPad Prism 8.0). *: $P < 0.05$; **: $P < 0.01$; ***: $P < 0.001$; N.S.: not significant.

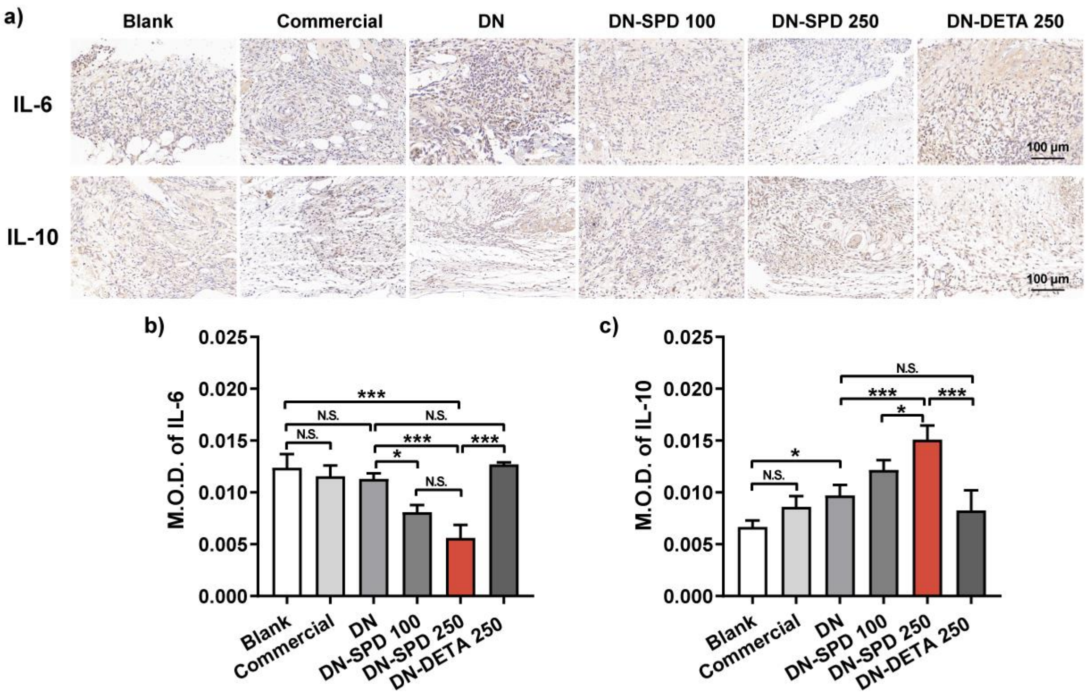

Figure S16. a) Immunohistochemical stainings of IL-6 and IL-10 on Day 7. **b, c)** Quantified M.O.D. of IL-6 **(b)** and IL-10 **(c)**. Data are presented as mean ± SD (n = 4) in **(b)** and **(c)**. Statistical significance was determined by using one-way ANOVA with Tukey's multiple comparisons (GraphPad Prism 8.0). *: *P* < 0.05; **: *P* < 0.01; ***: *P* < 0.001; N.S.: not significant.

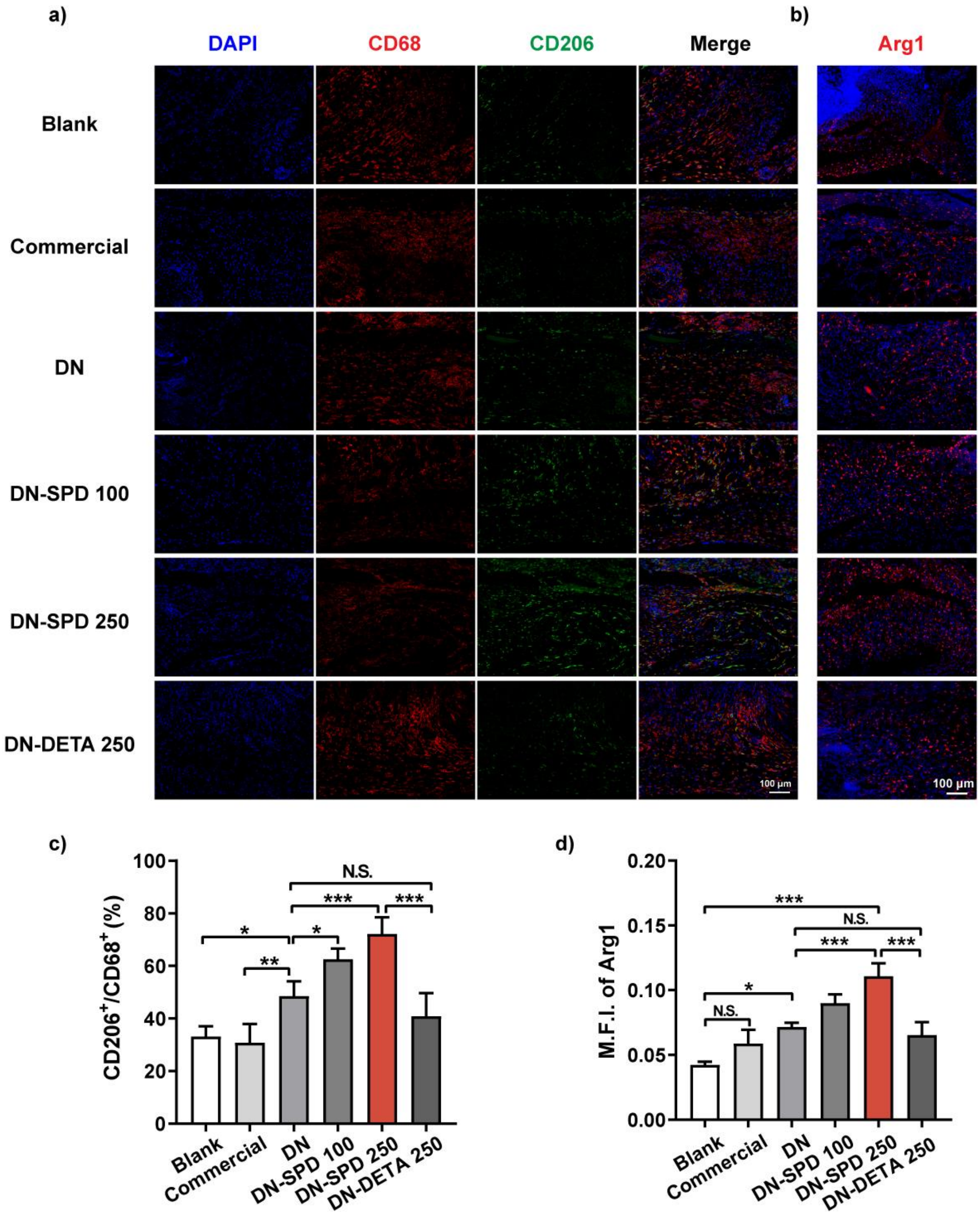

Figure S17. a, b) Double immunofluorescent staining of CD206/CD68 **(a)** and immunofluorescent staining of Arg1 **(b)** on Day 7. **c)** Quantified ratios of $CD206^+/CD68^+$. **d)** Quantified mean fluorescence intensities (M.F.I.) of Arg1 staining. Data are presented as mean ± SD (n = 4) in **(c)** and **(d)**. Statistical significance was determined by using one-way ANOVA with Tukey's multiple comparisons (GraphPad Prism 8.0). *: $P < 0.05$; **: $P < 0.01$; ***: $P < 0.001$; N.S.: not significant.

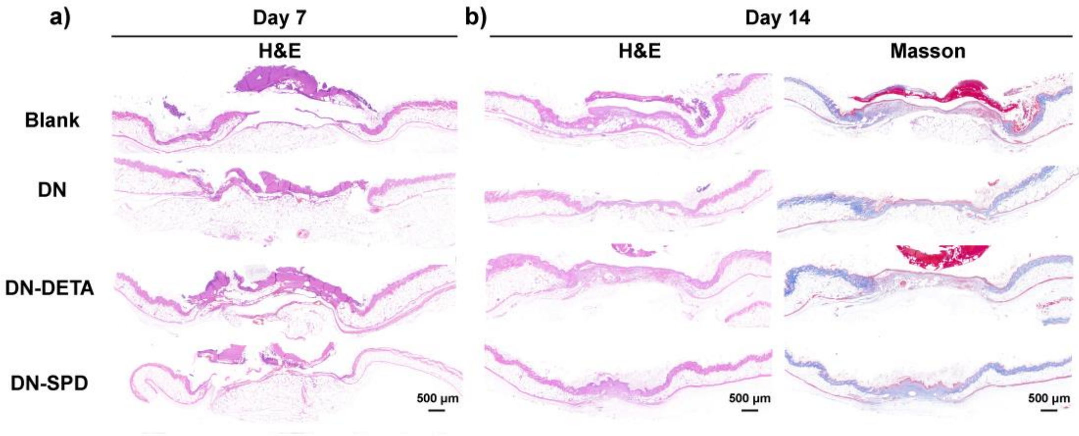

Figure S18. a) H&E staining of the wounds in *db*/*db* mice on Day 7. **b)** H&E and Masson’s trichrome stainings of the wounds in *db*/*db* mice on Day 14.

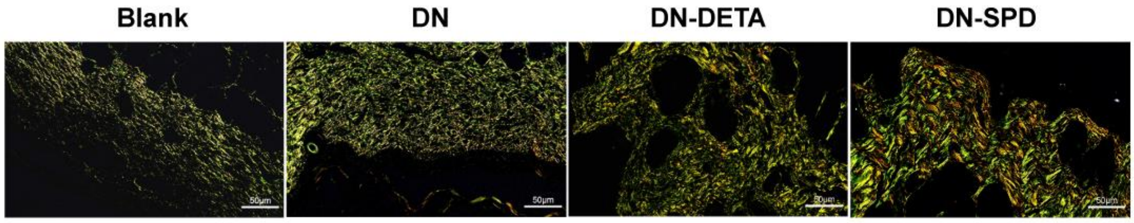

Figure S19. Sirius red staining of the wounds in *db*/*db* mice on Day 21.

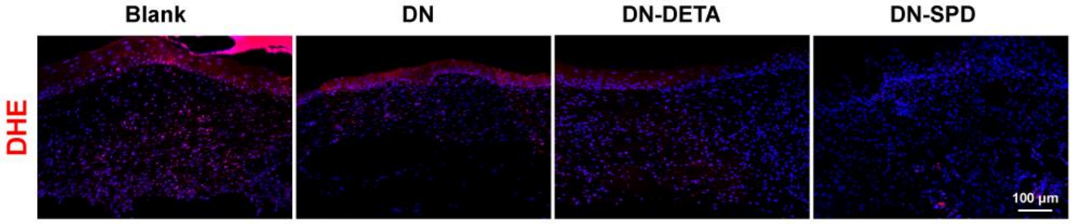

Figure S20. Immunofluorescent staining of ROS in the wounds of *db*/*db* mice on Day 14.

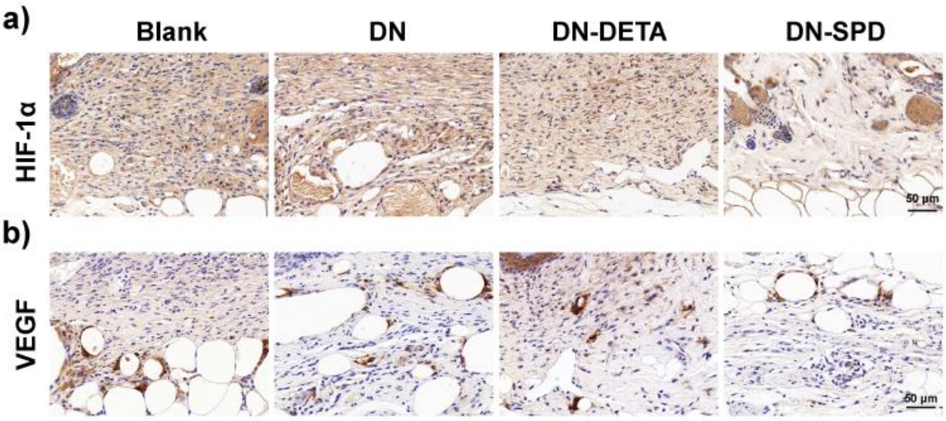

Figure S21. a, b) Immunohistochemical staining of HIF-1α **(a)** and VEGF **(b)** in the wounds of *db*/*db* mice on Day 21.

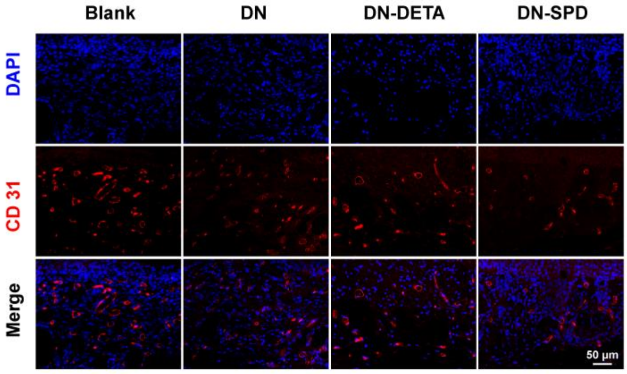

Figure S22. Immunofluorescent staining of CD31 in the wounds of *db*/*db* mice on Day 21.

3. Supplementary tables

Table S1. The sequences of primers used for RT-qPCR of macrophages *in vitro*

**Target**	**Primer sequence**	
TNF-α	Forward (F)	ATGAGCACAGAAAGCATGATCCG
	Reverse (R)	AGAGGCTGAGACATAGGCAC
iNOS	F	GGCTCCAGCATGTACCCT
	R	GCCCACTGAGTTCGTCCC
IL-1β	F	GAAATGCCACCTTTTGACAGTG
	R	TGGATGCTCTCATCAGGACAG
IL-6	F	GACTGATGCTGGTGACAACC
	R	AGACAGGTCTGTTGGGAGTG
CD86	F	GTGCCCATTTACAAAGGCTC
	R	CAGGTACTTGGCATTCACAC
TGF-β	F	TCCATCACTAGATCGCCCTT
	R	CTACCTTTGCCATTGCTT
IL-10	F	CAACATACTGCTAACCGACT
	R	GGCATCACTTCTACCA
CD206	F	CAAAGCCATGCTGTAGTACCG
	R	CCTTTTCATTTGTGCATGTGT
Arg1	F	CTGGCCTTTGTTGATGTCCCT
	R	CCCAGCACCACACTGACT
Actin	F	CTCCTGAGCGCAAGTACTCT
	R	TACTCCTGCTTGCTGATCCAC

Table S2. The sequences of primers used for RT-qPCR of tissues from subcutaneous implantation

Target	Primer sequence	
CD11b	Forward (F)	GTGTTTGCGTGTCAAGAAGAAGTAG
	Reverse (R)	ATGCGCCTGAGTATGCCGTT
CD68	F	TTGGATTCAAACAGGACCGAC
	R	TCTGCGCTGAGAATGTCCAC
TNF-α	F	CCAGGTTCTCTTCAAGGGACAA
	R	GGTATGAAATGGCAAATCGGCT
IL-6	F	GAGTTGTGCAATGGCAATTCTG
	R	ACGGAACTCCAGAAGACCAGAG
CD86	F	AGGACACGGGCTTGTATGATTG
	R	GGTTTCGGGTATCCTTGCTTAG
IL-1β	F	TGTGACTCGTGGGATGATGAC
	R	CCACTTGTTGGCTTATGTTCTGTC
IL-12	F	CACGCTACCTCCTCTTCTTGG
	R	AGCAGGTTTTGGGACTGGTTA
CD206	F	CATCTGCCAGCGACATAATAGC
	R	GGACACCAGGTTTCCTTTCAATC
CD31	F	ATCGGCAAAGTGGTCAAGAGAA
	R	TAGGAGGCGGTAAGTGATGGG
VEGF	F	GCAATGATGAAGCCCTGGAGT
	R	GGCTTTGTTCTATCTTTCTTTGGTC
GAPDH	F	CTGGAGAAACCTGCCAAGTATG
	R	GGTGGAAGAATGGGAGTTGCT

Table S3. Primary antibodies used in IHC

<table>
<tr><th>Antibody</th><th>Vendor</th><th>Catalog # & Dilution</th></tr>
<tr><td>Rabbit anti-TNF-α</td><td rowspan="4">Servicebio, Wuhan, China</td><td>GB11188, 1:200</td></tr>
<tr><td>Rabbit anti-IL-10</td><td>GB11534, 1:200</td></tr>
<tr><td>Rabbit anti-Collagen I</td><td>GB11022-3, 1:1000</td></tr>
<tr><td>Rabbit anti-Collagen III</td><td>GB111629, 1: 500</td></tr>
<tr><td>Rabbit anti-IL-1β</td><td rowspan="5">Abcam, UK</td><td>ab283818, 1:500</td></tr>
<tr><td>Rabbit anti-IL-12</td><td>ab131039, 1:500</td></tr>
<tr><td>Rabbit anti-IL-6</td><td>ab208113, 1:200</td></tr>
<tr><td>Rabbit anti-MPO</td><td>ab208670, 1:1000</td></tr>
<tr><td>Rabbit anti-HIF-1α</td><td>ab114977, 1:500</td></tr>
<tr><td>Rabbit anti-VEGFA</td><td>Proteintech, USA</td><td>19003-1-AP, 1:400</td></tr>
</table>

Table S4. Antibodies used in IF

Primary Antibody	Vendor	Catalog # & Dilution
Rabbit anti-CD11b	Servicebio, Wuhan, China	GB11058, 1:200
Rabbit anti -NF-kB p65		GB11997, 1:500
Rabbit anti-STAT1		GB111363, 1:500
Rabbit anti-MCP1		GB11199, 1:500
Rabbit anti-MMP8		GB11867, 1:200
Rabbit anti-CD68		GB113109, 1:500
Rabbit anti-IL-6		GB11117, 1:200
Rabbit anti-TNF-α		GB11188, 1:200
Rabbit anti-MMP9		GB12132, 1:200
Rabbit anti-CD163		GB13340, 1:200
Rabbit anti-CD86	Proteintech, USA	13395-1-AP, 1:500
Rabbit anti-CD206		18704-1-AP, 1:500
Rabbit anti-iNOS		18985-1-AP, 1:200
Rabbit anti-Arg1		16001-1-AP, 1:200
Rabbit anti-CD31	Abcam, UK	ab182981, 1:1000
Rabbit anti-α-SMA		ab124964, 1:500
**Secondary antibody**	**Vendor**	**Catalog # & Dilution**
Cy3-conjugated goat anti-rabbit IgG	Servicebio, Wuhan, China	GB21303, 1:300
Alexa Fluor 488-conjugated goat anti-rabbit IgG		GB25301, 1:400

4. List of supplementary videos

Video S1: the injectability of SN-SPD 250 hydrogel

Video S2: the self-healed SN-SPD 250 hydrogel under stretching

Video S3: the self-healed SN-SPD 100 hydrogel under stretching

Video S4: the 3D printing of SN-SPD 250 hydrogel before photo-crosslinking

Video S5: the *in situ* formation of DN-SPD 250 hydrogel on diabetic wound

5. Supplementary references

[1] J. Liu, M. Qu, C. Wang, Y. Xue, H. Huang, Q. Chen, W. Sun, X. Zhou, G. Xu, X. Jiang, *Small* **2022**, *18* (17), 2106172, https://doi.org/10.1002/smll.202106172.

[2] L. Mao, L. Wang, M. Zhang, M. W. Ullah, L. Liu, W. Zhao, Y. Li, A. A. Q. Ahmed, H. Cheng, Z. Shi, G. Yang, *Advanced Healthcare Materials* **2021**, *10* (14), 2100402, https://doi.org/10.1002/adhm.202100402.

[3] H. Lu, C. Tu, T. Zhou, W. Zhang, Y. Zhan, J. Ding, X. Wu, Z. Yang, W. Cao, L. Deng, C. Gao, F. Xu, *Chemical Engineering Journal* **2022**, *436*, 135130, https://doi.org/10.1016/j.cej.2022.135130.

[4] S. Wang, H. Zheng, L. Zhou, F. Cheng, Z. Liu, H. Zhang, L. Wang, Q. Zhang, *Nano Letters* **2020**, *20* (7), 5149, https://doi.org/10.1021/acs.nanolett.0c01371.

[5] W. Huang, S. Cheng, X. Wang, Y. Zhang, L. Chen, L. Zhang, *Advanced Functional Materials* **2021**, *31* (22), 2009189, https://doi.org/10.1002/adfm.202009189.

[6] a) J. Zhao, J.-y. Fu, F. Jia, J. Li, B. Yu, Y. Huang, K.-f. Ren, J. Ji, G.-s. Fu, *Advanced Functional Materials* **2023**, *33* (30), 2213993, https://doi.org/10.1002/adfm.202213993; b) J. Zhou, W. Liu, X. Zhao, Y. Xian, W. Wu, X. Zhang, N. Zhao, F.-J. Xu, C. Wang, *Advanced Science* **2021**, *8* (20), 2100505, https://doi.org/10.1002/advs.202100505.

[7] S. Jia, Z. Liu, S. Zhang, P. Liu, L. Zhang, S. H. Lee, J. Zhang, S. Signoretti, M. Loda, T. M. Roberts, J. J. Zhao, *Nature* **2008**, *454* (7205), 776, https://doi.org/10.1038/nature07091.
